# Probiotic-based nanoparticles for targeted microbiota modulation and immune restoration in bacterial pneumonia

**DOI:** 10.1093/nsr/nwac221

**Published:** 2022-10-16

**Authors:** Jieni Fu, Xiangmei Liu, Zhenduo Cui, Yufeng Zheng, Hui Jiang, Yu Zhang, Zhaoyang Li, Yanqin Liang, Shengli Zhu, Paul K Chu, Kelvin Wai Kwok Yeung, Shuilin Wu

**Affiliations:** School of Materials Science & Engineering, the Key Laboratory of Advanced Ceramics and Machining Technology by the Ministry of Education of China, Tianjin University, Tianjin 300072, China; School of Materials Science & Engineering, Peking University, Beijing 100871, China; School of Health Science & Biomedical Engineering, Hebei University of Technology, Tianjin 300401, China; School of Materials Science & Engineering, the Key Laboratory of Advanced Ceramics and Machining Technology by the Ministry of Education of China, Tianjin University, Tianjin 300072, China; School of Materials Science & Engineering, Peking University, Beijing 100871, China; School of Materials Science & Engineering, the Key Laboratory of Advanced Ceramics and Machining Technology by the Ministry of Education of China, Tianjin University, Tianjin 300072, China; Department of Orthopedics, Guangdong Provincial People's Hospital, Guangdong Academy of Medical Sciences, Guangzhou 510080, China; School of Materials Science & Engineering, the Key Laboratory of Advanced Ceramics and Machining Technology by the Ministry of Education of China, Tianjin University, Tianjin 300072, China; School of Materials Science & Engineering, the Key Laboratory of Advanced Ceramics and Machining Technology by the Ministry of Education of China, Tianjin University, Tianjin 300072, China; School of Materials Science & Engineering, the Key Laboratory of Advanced Ceramics and Machining Technology by the Ministry of Education of China, Tianjin University, Tianjin 300072, China; Department of Physics, Department of Materials Science and Engineering and Department of Biomedical Engineering, City University of Hong Kong, Hong Kong, China; Department of Orthopaedics & Traumatology, Li Ka Shing Faculty of Medicine, The University of Hong Kong, Hong Kong, China; School of Materials Science & Engineering, the Key Laboratory of Advanced Ceramics and Machining Technology by the Ministry of Education of China, Tianjin University, Tianjin 300072, China; School of Materials Science & Engineering, Peking University, Beijing 100871, China

**Keywords:** probiotic-based nanoparticles, immunocompetent primary bacterial pneumonia, immunocompromised secondary bacterial pneumonia, restoring host immunity

## Abstract

While conventional bacterial pneumonia mainly centralizes avoidance of bacterial colonization, it remains unclear how to restore the host immunity for hyperactive immunocompetent primary and immunocompromised secondary bacterial pneumonia. Here, probiotic-based nanoparticles of OASCLR were formed by coating chitosan, hyaluronic acid and ononin on living *Lactobacillus rhamnosus*. OASCLR nanoparticles could effectively kill various clinic common pathogens and antibacterial efficiency was >99.97%. Importantly, OASCLR could modulate lung microbiota, increasing the overall richness and diversity of microbiota by decreasing pathogens and increasing probiotic and commensal bacteria. Additionally, OASCLR could target inflammatory macrophages by the interaction of OASCLR with the macrophage binding site of CD44 and alleviate overactive immune responses for hyperactive immunocompetent pneumonia. Surprisingly, OASCLR could break the state of the macrophage's poor phagocytic ability by upregulating the expression of the extracellular matrix assembly, immune activation and fibroblast activation in immunocompromised pneumonia. The macrophage's phagocytic ability was increased from 2.61% to 12.3%. Our work provides a potential strategy for hyperactive immunocompetent primary and immunocompromised secondary bacterial pneumonia.

## Teaser

The probiotic-based nanoparticles could effectively treat immunocompetent primary and immunocompromised secondary bacterial pneumonia by restoring host immunity.

## INTRODUCTION

Bacterial pneumonia is a significant cause of childhood morbidity and mortality worldwide [1]. The 2019 Global Burden of Diseases study showed that there were 68.46 million episodes of bacterial pneumonia worldwide in 2019, contributing to 0.34 million mortalities [2,3]. More importantly, murine alveolar macrophage presents poor phagocytic capacity for several weeks after resolution of primacy pneumonia [4] and secondary bacterial pneumonia is usually more severe than primary bacterial pneumonia [5]. Bacterial pneumonia is closely related to the imbalance of the lung microbiota and successive dysregulation of lung immune responses [2,6]. Traditional medical strategies for pneumonia have concentrated on avoiding bacterial colonization and various antimicrobial agents are taken advantage of to combat bacterial infection, such as antibiotics, antibacterial vaccines, antibodies, antibody–drug conjugates, phages, antimicrobial peptides, phage, Chinese medicine small molecule materials and so on [2,7–14]. Antibiotics are the mainstay for pneumonia therapy during these antimicrobial agents [2]. On the one hand, the overuse of antibiotics leads to the rapid emergence of antibiotic-resistant bacteria [15,16]. The development of a new antibiotic would take >10 years and cost >1.5 billion pounds [17]. On the other hand, antibiotics can weaken the phagocytic killing in immune cells by inhibiting respiratory activity [18,19] and destruct the lung microbiome [20]. Therefore, there is an imperative need to devise better therapies to restore immune response and enhance the host's resistance to infection in primary and second bacterial pneumonia.

We designed a kind of nanoparticle of OASCLR (living *Lactobacillus rhamnosus* (LR) modified with chitosan (CS), hyaluronic acid (HA) and ononin). As one of the most common living probiotics found in the human body, LR can modulate the immune response in hyperactive immunocompetent and immunocompromised hosts [21], and resist microbes [22]. However, LR is a rod-shaped, microaerophilic and facultatively anaerobic bacterium [23]. The normal oxygen pressure in the lung would influence the viability of microaerophilic bacteria [24]. Microencapsulation is an alternative strategy for preventing probiotics from being damaged [25,26]. CS, as a cationic polymer, is commonly used in oral delivery applications and exhibits unique mucoadhesive properties with excellent biocompatibility [27]. CS can be layered on LR via electrostatic interactions. In addition, HA, as a glycosaminoglycan biopolymer [28], is commonly found in the extracellular matrix [29] and HA has immunomodulatory ability by regulating macrophage [30] and CD4^+^ T (T_reg_) cells [31]. However, HA would have a rapid turnover under harsh oxidative conditions [29]. Ononin, as an isoflavone component in traditional Chinese medicines, can be found in *Astragalus membranaceus, Glycyrrhiza uralensis, Hedysarum* and *Pueraria lobata* [32]. Ononin has robust reactive oxygen species (ROS)-scavenging [33] anti-inflammatory [34] and anti-oxidant properties [35]. More importantly, ononin can promote the growth of LR and inhibit the growth of pathogen bacteria [36].

**Figure 1.**
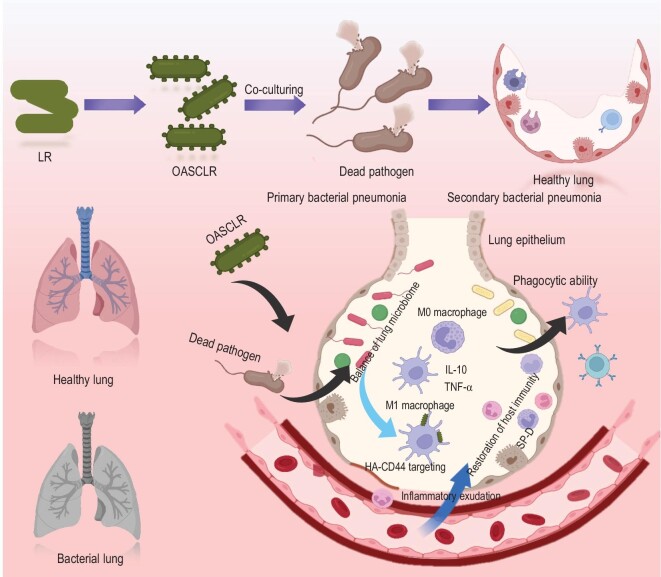
OASCLR exerted therapeutic effects against primary pneumonia and secondary pneumonia. Schematic illustration of OASCLR against primary and secondary pneumonia. First, OASCLR inherited the innate poverty of LR competing with various pathogens and OASCLR killed various pathogens. On the other hand, OASCLR altered the composition of the lung microbiome. In addition, OASCLR targeted M1 macrophages by HA–CD44 interactions and modulated the overactivated immune response of macrophages. Specifically, OASCLR inhibited the inflammatory response in primary pneumonia. More importantly, OASCLR enhanced the phagocytosis ability of the macrophage in secondary pneumonia by downregulating the expression of SP-D.

Considering the low bioactivity of LR in the ROS environment, the designed CS/HA–ononin shell could prevent LR from oxygen damage and allow OASCLR nanoparticles targeting at pro-inflammatory macrophages by the interaction of HA with CD44 (the receptor macrophage). Moreover, the LR core could permit the OASCLR nanoparticles to kill pathogenic bacteria and potentially modulated lung microbiota in primary bacterial pneumonia. Unexpectedly, our studies disclosed that OASCLR nanoparticles also had great treatment efficacy against secondary bacterial pneumonia. OASCLR nanoparticles could upregulate the expression of extracellular matrix assembly, immune activation and fibroblast activation based on ribonucleic acid (RNA) sequencing. In addition, OASCLR nanoparticles could inhibit the surfactant proteins (SP)-D expression for macrophages. These all were related to the improved function of the phagocytic ability of macrophages, which could overcome the state of lung immunoparalysis in secondary pneumonia (Fig. 1). Our study demonstrated that OASCLR nanoparticles had effective treatment efficacy for primary and secondary bacterial pneumonia.

## RESULTS AND DISCUSSION

### Preparation, characterization, viability and resistance of OASCLR nanoparticles

**Figure 2.**
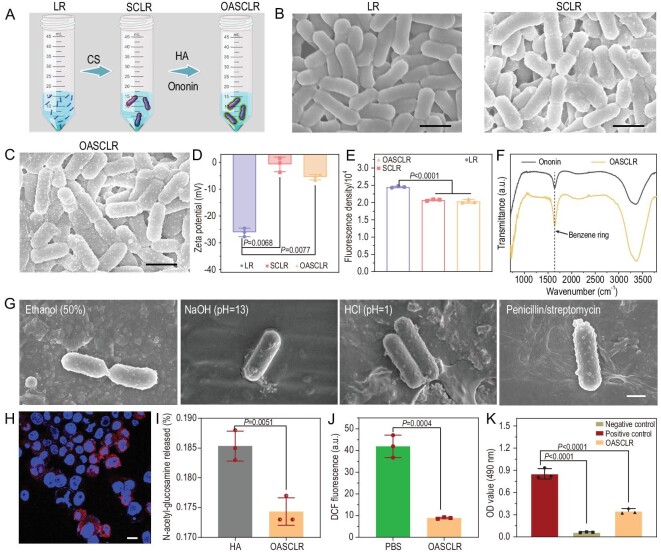
Preparation, characterization, viability and resistance of OASCLR nanoparticles. (A) Schematic illustration of the formation of SCLR and OASCLR. (B) SEM images of uncoated LR and SCLR. Scale bar, 500 nm. (C) SEM image of OASCLR. Scale bar, 500 nm. (D) The value of the zeta potential of different samples (LR, SCLR and OASCLR) (*n* = 3 biologically independent samples). (E) The fluorescence density of different samples (LR, SCLR and OASCLR) (*n* = 3 biologically independent samples). (F) The FTIR plots of OASCLR and ononin. (G) The SEM images of OASCLR cultured in ethanol (50%, v/v), NaOH (pH = 13), HCl (pH = 1) and penicillin/streptomycin at 37°C for 2 h. Scale bar, 0.5 μm. (H) Confocal microscopy image of macrophages pre-treated for 24 h with LPS (100 ng/mL), followed by 1 h of incubation with OASCLR Cy 5.5 (10^7^ CFU/mL). The cell nuclei were stained using Hoechst 33342 (blue) and OASCLR using Cy 5.5 (red). Scale bar, 10 μm. (I) Generation of *N*-acetyl-glucosamine after treatment of 1 mg/mL HA or OASCLR with 10^9^ CFU (*n* = 3 biologically independent samples). (J) Fluorescence signals of DCF oxidized from DCFDA (50 μM) by peroxy radicals generated from 1 mM of AAPH at 37°C in the presence of OASCLR (10^9^ CFU) or PBS (*n* = 3 biologically independent samples). (K) Viability of RAW 264.7 cells was measured using MTT assay after overnight treatment with OASCLR (10^9^ CFU) or PBS in the presence of 100 μM H_2_O_2_, or PBS without 100 μM H_2_O_2_ (*n* = 3 biologically independent samples). Data are presented as mean ± standard deviation (SD). (D, E and K) Data were analysed using one-way ANOVA with multiple comparison test. (I and J) Data were analysed using *t*-test.

As illustrated in Fig. 2A, LR was coated with CS to form SCLR in phosphate-buffered saline (PBS). HA and ononin were further used to coat SCLR to create OASCLR in PBS. Scanning electron microscopy (SEM) images displayed a clear extra outer shell on SCLR and OASCLR compared with LR (Fig. 2B)and C). The lengths of LR, SCLR and OASCLR were 1.15 ± 0.30, 1.20 ± 0.14 and 1.32 ± 0.23 μm, respectively. The widths of LR, SCLR and OASCLR were 0.47 ± 0.03, 0.49 ± 0.03 and 0.51 ± 0.04 μm, respectively. Transmission electron microscopy images further demonstrated this point (Supplementary Fig. S1). These results demonstrated that the size of LR, SCLR and OASCLR was stable. It is generally known that Gram-positive bacterial cell walls have negative charges due to the presence of teichoic acids [37]. CS and HA are positively and negatively charged polymers, respectively. The zeta potential measurement showed that the zeta potential values of LR, SCLR and OASCLR were –26.09 ± 0.91, –0.75 ± 1.56 and –5.51 ± 0.58 mV (Fig. 2D), suggesting that the forming process was mainly related to electrostatic interaction. The metabolic substance of the bacteria would be detected using the AlamarBlue (resazurin) dye [38]. As shown in Fig. 2E, the coating almost did not influence bacterial viability. Moreover, LR, SCLR and OASCLR underwent bacterial live/dead staining to visualize the bioactivity of LR after coating. LR in SCLR and OASCLR all showed green fluorescence (live bacteria) and no red fluorescence (dead bacteria), suggesting that LR was still live after coating (Supplementary Fig. S2). Fourier transform infrared spectroscopy (FTIR) was further used to assess whether ononin was integrated into the shell of OASCLR. There were peaks at 1643, corresponding to the benzene ring (Fig. 2F). The result demonstrated that there was ononin in OASCLR. Meanwhile, ononin and dead LR were labeled using fluorescein isothiocyanate (FITC) and propidium iodide, respectively. Supplementary Fig. S3 shows that green fluorescence was localized around red fluorescence, demonstrating that ononin was distributed around the surface of LR. The amount of CS, HA and ononin loaded onto the surface of LR was characterized using a UV–Vis spectrophotometer (Supplementary Fig. S4). The OASCLR loaded 0.41 mg CS, 0.16 mg HA and 0.31 μg ononin when the LR was 10^9^ colony forming units (CFU). The CS and CS/HA–ononin shells were coated on LR and the experiment was further performed to characterize whether those shells could protect LR from harsh environmental assaults. Ethanol (50%, v/v), NaOH (pH = 13), HCl (pH = 1) and antibiotic (penicillin/streptomycin) was used to respectively simulate ethanol, strong alkalinity, strong acidity and antibacterial chemicals. Ethanol could directly kill bacteria by dehydrating and denaturing proteins [26]. LR, SCLR and OASCLR were incubated in ethanol (50%, v/v), NaOH (pH = 13), HCl (pH = 1) and antibiotic (penicillin/streptomycin) solutions for 2 h, respectively. The bacterial morphologies of LR, SCLR and OASCLR after different treatments were detected using SEM. In the case of the SCLR and OASCLR groups, the images showed that the bacterial morphology still was rod-shaped (Fig. 2G)and Supplementary Fig. S5). In contrast, the bacteria in the LR group appeared aberrant (marked by red arrows) (Supplementary Fig. S5). In addition, LR, SCLR and OA SCLR were incubated in a simulated pulmonary environment pH (pH = 6) for 2 h. The LR group showed that the integrity of the bacterial membrane was disrupted at a certain level but the SCLR and OASCLR groups exhibited normal shapes (Supplementary Fig. S6). The bacterial live/dead fluorescence staining showed similar results (Supplementary Fig. S7). The results demonstrated that the coating membranes improved the resistance of SCLR or OASCLR against severe environments.

HA could target specifically the pro-inflammatory M1 macrophage via interacting with the CD44 receptor on its membrane [28]. Cell fluorescence assay was further performed to assess whether OASCLR could target M1 macrophages. The macrophages tended to polarize into M1-type and M2-type phenotypes after incubating with lipopolysaccharides (LPS) and IL-4, respectively (Supplementary Fig. S8). OASCLR and OSCLR were labeled using Cy 5.5 (red) and Hoechst 33342 (blue) was used for M1 macrophages (Fig. 2H)and Supplementary Fig. S9A). When OASCLR was incubated with M0/M1/M2 macrophages for 2 h, OASCLR was obviously attached to the surface of the pro-inflammatory M1 macrophage (Fig. 2H)and Supplementary Fig. S9A). In M1-type macrophages pre-treated with either anti-CD44 antibody or incubated with OSCLR (free HA), the red signal was abrogated (Supplementary Fig. S9B). The results confirmed that OASCLR could target the M1 macrophage through CD44–HA interaction. However, HA could be degraded by hyaluronidase to produce *N*-acetyl-glucosamine [28]. The content of *N*-acetyl-glucosamine was further monitored to evaluate whether ononin could protect HA from hyaluronidase, when incubating hyaluronidase with HA or OASCLR. As shown in Fig. 2I, the content of *N*-acetyl-glucosamine in the OASCLR group was obviously lower than that in the pure HA group, suggesting that ononin addition enhanced the resistance ability of OASCLR against hyaluronidase degradation. The condition in primary pneumonia is a harsh oxidative environment. 2,2′-azobis (2-amidinopropane) (AAPH) is a water-soluble azo compound and it is used extensively as a peroxy radical generator. The ROS-scavenging ability of OASCLR was measured via a 2′-7′-dichlorodihydrofluorescein diacetate (DCFH-DA) fluorescent probe, which is widely used for directly measuring the content of ROS [39]. DCFH-DA would be oxidized into 2′,7′-dichlorofluorescein (DCF) when DCFH-DA was exposed to ROS and the fluorescence signal would increase. As shown in Fig. 2J, the DCF fluorescence intensity in the OASCLR group was obviously lower than that in the PBS group, suggesting that OASCLR had ROS-scavenging ability. To further characterize the protective effect of OASCLR on cells against ROS-mediated cytotoxicity, the viability of the RAW 264.7 cells was measured using a 3-[4,5-dimethylthiazol-2-yl]-2,5 diphenyl tetrazolium bromide (MTT) assay after 24 h of treatment with PBS or OASCLR in the presence of 100 μM of H_2_O_2_. Compared with the cell viability in the negative control group (cells treated with PBS in the presence of 100 μM of H_2_O_2_), the cell viability in the OASCLR group was significantly enhanced, demonstrating that OASCLR succeeded in protecting RAW 264.7 cells from ROS (Fig. 2K).

### Biocompatibility and immune regulation of OASCLR

**Figure 3.**
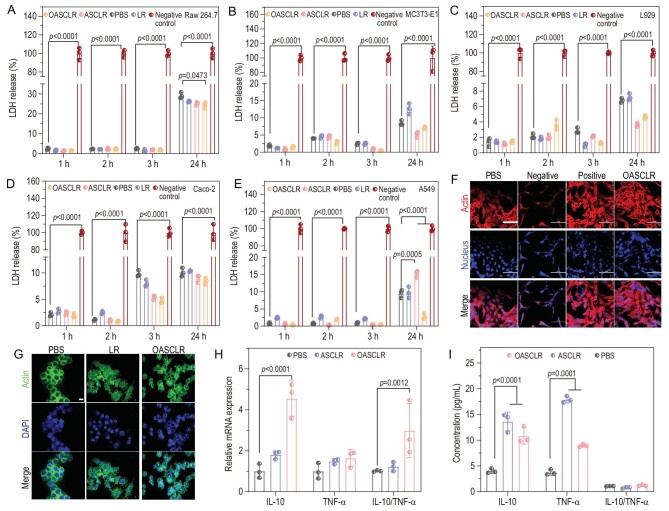
Biocompatibility and immune regulation of OASCLR *in vitro*. (A–E) Viability of (A) RAW 264.7 cells, (B) MC3T3-E1 cells, (C) L929 cells, (D) Caco-2 cells and (E) A549 cells measured using LDH assay after 1, 2, 3 and 24 h of treatment with PBS, LR, ASCLR, OASCLR and LDH release reagent (negative control) (*n* = 3 biologically independent samples). (F) The fluorescence images of A549 cells. PBS group was A549 cells treated with PBS without H1N1. Negative, positive and OASCLR groups were A549 cells treated with PBS, oseltamivir phosphate (100 mg/mL) and OASCLR (10^7^ CFU/mL), respectively, in the presence of H1N1. Scale bar, 100 μm. (G) The fluorescence images of RAW 264.7 treated with PBS, LR and OASCLR. Scale bar, 10 μm. (H) qPCR analysis of gene IL-10 and gene TNF-α expressions following 48 h of treatment with PBS, ASCLR and OASCLR (*n* = 3 biologically independent samples). (I) Concentration of protein IL-10 and protein TNF-α expressions analysed using ELISA (*n* = 3 biologically independent samples). Data are presented as mean ± standard deviation (SD). (A–E, H and I) Data were analysed using two-way ANOVA with multiple comparison test.

LR, ASCLR and OASCLR all contained living bacteria, so a cell cytotoxicity assay of those samples needed to be performed. The cell viability was analysed through a lactate dehydrogenase (LDH) assay. LDH would be rapidly released into the cell culture medium upon cell membrane damage, so the content of the LDH release was related to the cell cytotoxicity. Various cells, such as RAW 264.7 macrophage cells, MC3T3-E1 osteoblast cells, L929 fibroblast cells, Caco-2 epithelial cells and A549 epithelial cells, were chosen to investigate the cell cytotoxicity of LR, ASCLR and OASCLR. Compared with the PBS group, the percentage of LDH release in the OASCLR group was lower for all cells at 1, 2, 3 and 24 h, respectively (Fig. 3A–E). Those results suggested that OASCLR had outstanding biocompatibility for these cells. In addition, A549 cells were chosen to evaluate the antivirus ability of OASCLR against the influenza A (H1N1) virus. The intracellular actin and cell nucleus were stained using tetra-methyl-5,6-isothiocyanate (TRITC)-labeled phalloidin (red) and 4′,6-diamidino-2-phenylindole (DAPI, blue), respectively (Fig. 3F). As for cells in the PBS, positive and OASCLR groups, the A549 cells showed spread morphologies. In contrast, the cells in the negative group displayed shrunk morphologies. The result indicated that OASCLR successfully protected A549 cells from H1N1 virus assault. On the one hand, the capsid protein of the virus could be altered by the acidic pH caused by LR, which would prevent cell adhesion. On the other hand, the bacteriocin and hydrogen peroxide secreted by LR could inhibit viral replication by preventing the virus from entering host cells [40].

Macrophages can function in innate and adaptive immunity [41] and pro-inflammatory M1 macrophages would cause serious side effects to the lung due to the cytokine storm in pneumonia [11]. OASCLR exhibited significant ROS-scavenging activity from the above data, which led us to ask whether OASCLR could regulate the inflammatory response of macrophages. RAW 264.7 cells were incubated using PBS, LR or OASCLR for 24 h, respectively. FITC-labeled phalloidin (green) and DAPI (blue) were used to visualize the intracellular actin and cell nucleus, respectively (Fig. 3G). The cell morphology was observed using confocal fluorescence microscopy. The RAW 264.7 cells treated with PBS showed round morphologies. In contrast, cells in the LR group had short filopodia that extended from the cell, which was related to the macrophage's phagocytosis behavior towards LR. Meanwhile, the cells in the OASCLR group displayed fusiform morphologies, indicating that macrophages had the potential to differentiate into anti-inflammatory M2-type macrophages. The result revealed that OASCLR could evade immune attack through the CS/HA–ononin shell and be effectively cleared after the loss of encapsulation. To further analyse the influence of OASCLR on the macrophage polarization state, interleukin 10 (IL-10; marker for anti-inflammatory M2-type macrophage) [42] and tumor necrosis factor-alpha (TNF-α; marker for pro-inflammatory M1-type macrophage) [43] were chosen for further characterization. The ratio value between IL-10 and TNF-α could express the polarization state of the macrophages, and a larger value represented a stronger polarization tendency of macrophages towards M2. The gene expressions of IL-10 and TNF-α were detected using quantitative real-time PCR (qRT-PCR) (Fig. 3H). As shown in Fig. 3H, compared with the PBS group, the ratio value between IL-10 and TNF-α in the OASCLR group and ASCLR was 2.77 and 1.22, respectively. The ratio value between IL-10 and TNF-α in the OASCLR group was bigger than that in the ASCLR group. On the other hand, an enzyme-linked immunosorbent assay (ELISA) was performed to measure the protein expressions of IL-10 and TNF-α (Fig. 3I). Figure 3I)shows that the ratio value between IL-10 and TNF-α in the OASCLR, ASCLR and PBS groups was 1.21, 0.76 and 1.07, respectively. The ratio value between IL-10 and TNF-α in the OASCLR group was bigger than those in the ASCLR and PBS groups. Those data suggested that OASCLR could polarize macrophages into M2 macrophages at the gene and protein levels.

### OASCLR could avoid bacterial colonization

**Figure 4.**
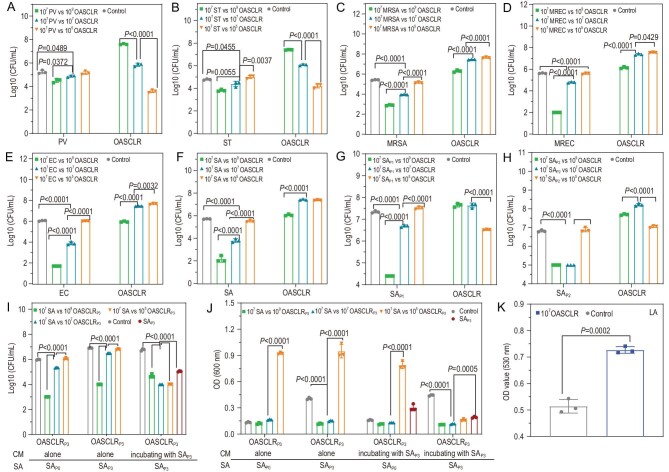
OASCLR could avoid bacterial colonization. (A–F) The CFU value of OASCLR and (A) PV, (B) ST, (C) MRSA, (D) MREC, (E) EC and (F) SA after co-culture of various concentrations of OASCLR with PV, ST, MRSA, MREC, EC and SA at 37°C for 24 h, respectively. (G) The CFU value of OASCLR and SAP1 after incubating different concentrations of OASCLR with SAP1 at 37°C for 24 h. (H) The CFU value of OASCLR and SAP2 after incubating different concentrations of OASCLR with SAP1 at 37°C for 24 h. The control was (A) PV, (B) ST, (C) MRSA, (D) MREC, (E) EC, (F) SA, (G) SAP1 and (H) SAP2 culturing in MRS broth for 24 h. (I) The CFU value of SAP0 and SAP3 after treating different CM. The left control was SAP0 culturing in the CM of OASCLRP3 culturing alone for 24 h. The middle control was SAP3 culturing in the CM of OASCLRP3 culturing alone for 24 h. The right control was SAP3 culturing in the CM of OASCLRP3 co-culturing with SAP3 for 24 h. (J) The optical density (OD) value of SAP0 or SAP3 after treating different CM. The first control on the left was SAP0 culturing in the CM of OASCLRP3 culturing alone for 24 h. The second control on the left was SAP3 culturing in the CM of OASCLRP3 culturing alone for 24 h. The third control on the left was SAP0 culturing in the CM of OASCLRP3 co-culturing with SAP3 for 24 h. The fourth control was SAP3 culturing in the CM of OASCLRP3 co-culturing with SAP3 for 24 h. (K) The concentration of lactic acid (LA) of different groups (control and 10^7^ OASCLR) (*n* = 3 biologically independent samples). Data are presented as mean ± standard deviation (SD). Data were analysed using two-way ANOVA with multiple comparison test.

The core of OASCLR was LR, so OASCLR inherited the innate function of LR competing with various pathogens. The probiotic could inhibit pathogen growth by secretion of antibacterial substances and organic acid or competing for resources and space [44]. On the other hand, ononin could enhance the growth of LR but inhibit the growth of pathogens (Supplementary Figs S10 and S11). *Proteusbacillus vulgaris* (PV), *Salmonella typhimurium* (ST), Gram-positive methicillin-resistant *Staphylococcus aureus* (MRSA), multi-resistant *Escherichia coli* (MREC), *E. coli* (EC) and *S. aureus* (SA) are several kinds of clinically common pathogens. Those pathogens are responsible for most infections, including pulmonary infections [45]. The numbers of pathogen bacteria and OASCLR are displayed in Fig. 4A–F and Supplementary Figs S12–S17 after co-culture of PV, ST, MRSA, MREC, EC and SA with different concentrations of OASCLR for 24 h. OASCLR (10^9^ colony-forming units per milliliter (CFU/mL)) exhibited significant antibacterial efficiencies against PV (81.77 ± 6.01%), ST (89.01 ± 2.20%), MRSA (99.70 ± 0.03%), MREC (99.98 ± 0.01%), EC (99.99 ± 0.01%) and SA (99.97 ± 0.02%). As for OASCLR at a concentration of 10^7^ CFU/mL, they killed 57.49 ± 7.81% of PV, 55.44 ± 19.91% of ST, 96.54 ± 0.16% of MRSA, 85.37 ± 1.71% of MREC, 99.29 ± 0.25% of EC and 98.73 ± 0.48% of SA. However, 10^5^ CFU/mL OASCLR exhibited weak bacteria-killing efficiencies of 15.14 ± 27.55%, –72.20 ± 46.67%, 46.81 ± 7.19%, 7.61 ± 12.96%, 6.86 ± 3.68% and 36.14 ± 11.54% against PV, ST, MRSA, MREC, EC and SA, respectively. These data demonstrated that the antibacterial efficiency of OASCLR was positively related to the amount of OASCLR. Supplementary Table S1 further shows the minimum bactericidal concentrations (MBCs) of LR and OASCLR against PV, ST, MRSA, MREC, EC and SA. The MBCs of LR against PV, ST, MRSA, MREC, EC and SA were 1.25 × 10^8^, 1 × 10^9^, 1 × 10^9^, 1.95 × 10^6^, 1.95 × 10^6^ and 2.5 × 10^8^ CFU/mL, respectively. The MBCs of OASCLR against PV, ST, MRSA, MREC, EC and SA were 2 × 10^9^, 4 × 10^9^, 1 × 10^9^, 1.25 × 10^8^, 1.25 × 10^8^ and 5 × 10^8^ CFU/mL, respectively.

Next, the group (10^7^ CFU/mL pathogens vs 10^7^ CFU/mL OASCLR) was further chosen to investigate the interaction between the pathogen and LR. The bacterial value of PV, ST, MRSA, MREC, EC and SA in the control group (the pathogen of 10^7^ CFU/mL culturing in Man–Rogosa–Sharpe (MRS) broth for 24 h, pathogens were PV, ST, MRSA, MREC, EC and SA) was >10^7^ pathogens vs 10^7^ in the OASCLR group (the pathogen of 10^7^ CFU/mL culturing with LR of 10^7^ CFU/mL in MRS broth for 24 h, pathogens were PV, ST, MRSA, MREC, EC and SA) (Fig. 4A–F and Supplementary Figs S12–S17). The result indicated that the presence of LR would influence on the growth of pathogens. Meanwhile, the growth of OASCLR itself without pathogens was also detected and OASCLR could grow to ∼10^9^ CFU/mL after 24 h of culturing (Supplementary Fig. S18). However, when different concentrations of OASCLR were incubated with MRSA, MREC, EC, SA, PV and ST, the amount of LR in OASCLR did not grow to 10^9^ CFU/mL (Fig. 4A–F and Supplementary Figs S12–S17). These data suggested that PV, ST, MRSA, MREC, EC and SA also could influence the growth of LR.

Importantly, it is generally known that bacterial resistance would develop after several successive antibacterial cycles [46]. OASCLR was further used in the following antibacterial experiments against different passages of SA. The survived SA after the first antibacterial cycle of incubating OASCLR with SA was named SAP1. As shown in Fig. 4G)and Supplementary Fig. S19, OASCLR (10^7^ CFU/mL) killed 77.54 ± 2.60% of SAP1 compared with the control group (SAP1 incubated in MRS broth without OASCLR for 24 h), when the concentration of OASCLR was 10^7^ CFU/mL. The survived SA after the second antibacterial cycle of incubating OASCLR with SA was named SAP2. As shown in Fig. 4H)and Supplementary Fig. S20, OASCLR (10^7^ CFU/mL) killed 98.98 ± 0.88% of SAP2 compared with the control group (SAP2 were incubated in MRS broth without OASCLR for 24 h). Also, 10^9^ CFU/mL OASCLR showed great antibacterial efficiency. If different bacteria grew in the same space, competition for space and resources would occur [47]. So the antibacterial efficiency of OASCLR (10^9^ CFU/mL) was better than 10^7^ CFU/mL of OASCLR. These data demonstrated that bacterial resistance was not developed during the process of several successive antibacterial cycles.

The antibacterial mechanism of OASCLR against pathogens was next investigated. The survived LR after the third cycle of incubating OASCLR with SA was coated with CS, HA and ononin and then it was named OASCLRP3. The culturing medium (CM) was extracted from incubating OASCLRP3 alone or incubating OASCLRP3 with different passages of SA to assess its antibacterial efficiencies against SAP0 or SAP3 (Fig. 4I)and J, and Supplementary Fig. S21). As shown in Fig. 4I)and Supplementary Fig. S21, the CM extracted from incubating 10^9^ CFU/mL of OASCLRP3 alone showed great antibacterial efficiency against SAP0 or SAP3. But 10^5^ and 10^7^ CFU/mL of OASCLR behaved with weak antibacterial efficiency against SAP0 or SAP3. The antibacterial effect was positively related to the amount of OASCLRP3. The result demonstrated that the excreta from OASCLR had a certain antibacterial effect against SA. Next, the antibacterial efficiency of secretion from SA or SA/OASCLR against SA was further analysed. Unexpectedly, the bacterial number value was lower than in culturing in CM extracted from incubating pure SAP3 in MRS broth for 24 h compared with the control group (incubating SAP3 in MRS broth for 24 h), suggesting that CM, extracted from incubating pure SAP3 in MRS broth for 24 h, also showed a certain antibacterial effect against SAP3 (Fig. 4I)and J). The result suggested that CM of SAP3 could inhibit the growth of SAP3. The growth and survival of SA are dependent on environmental factors. The component of CM would vary with the growth of SA. Available substrates would decrease and adverse catabolites would be produced [48]. Those changes could inhibit the growth of SA. Finally, we characterized the antibacterial efficiency of secretion from medium co-culturing SAP3 with different concentrations of OASCLRP3 against SAP3 (Fig. 4I)and J). When the concentration of OASCLRP3 was 10^5^ and 10^7^ CFU/mL, the antibacterial efficiency of CM extracted from medium co-culturing SAP3 with different concentrations of OASCLRP3 against SAP3 was obviously better than CM of different concentrations of OASCLRP3 after 24 h of culturing alone. The result might indicate that some extra substances produced during the process of incubating OASCLRP3 with SAP3 might take part in the antibacterial process. These results indicated that the antibacterial ability of OASCLR was not only from the secretion of LR but also from the excreta of SA. The content of lactic acid (LA) was further analysed after 10^7^ CFU/mL of OASCLR was cultured for 12 h. As shown in Fig. 4K, the content of LA in the 10^7^ CFU/mL of OASCLR group was obviously higher than that in the control group (MRS broth), suggesting that LA was produced during the culturing process of OASCLR. LR could produce LA through carbohydrate metabolism and the organic acid could decrease the pH of the environment *in situ*. Lower pH discouraged the growth of pathogens [49].

### OASCLR modulated lung microbiota and alleviated inflammatory response in hyperactive immunocompetent primary bacterial pneumonia

**Figure 5.**
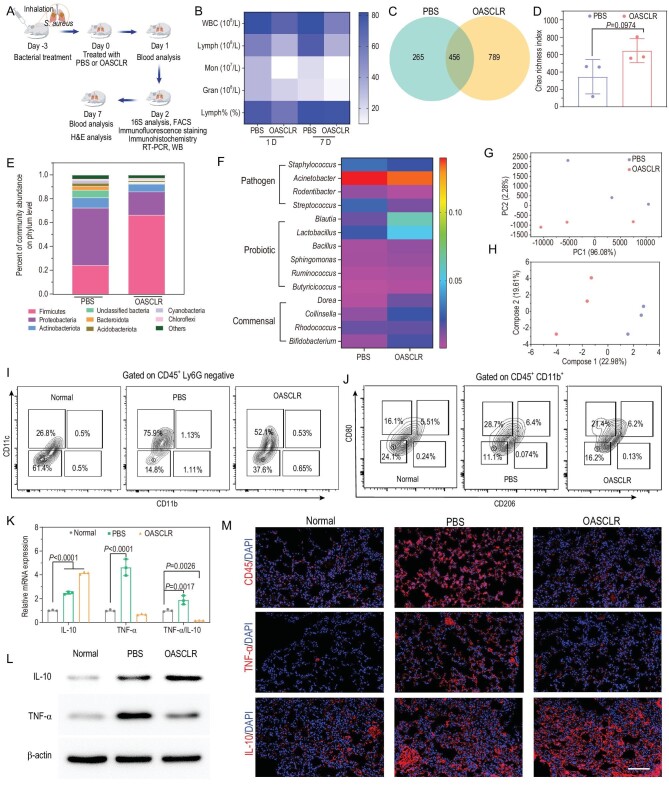
OASCLR modulated lung microbiota and alleviated inflammatory response in hyperactive immunocompetent primary bacterial pneumonia. (A) Schematic diagram of the experimental outline of primary pneumonia with SA. (B) Blood routine test results of different groups (PBS and OASCLR) on Days 1 and 7. (C) Venn diagram of different groups (PBS and OASCLR). (D) The Chao richness index of two groups for the estimation of microbial community diversity. (E) Distribution of microbial communities of different groups (PBS and OASCLR) at the phylum level. (F) Heat map between the PBS and OASCLR groups classified in OTUs. (G) The PCA plot of two groups. (H) Partial least squares discriminant analysis (PLS-DA) between the PBS and OASCLR groups. (I) FACS analysis of lung tissue of the normal, PBS and OASCLR groups. Subsets gated on CD45^+^ LY6G^−^ were analysed based on the expression of CD11b and CD11c as inflammatory monocytes (CD11b^high^, CD11c^neg^) and alveolar macrophages (CD11c^high^, CD11b^neg^). (J) M1- and M2-polarization phenotypes of CD45^+^ CD11b^+^ cells in different groups (normal, PBS and OASCLR groups) by measuring the expression of CD206 and CD80. (K) qRT-PCR analysis of gene IL-10 and gene TNF-α expressions in lung tissue of mice. (L) The protein expression of M1 marker (TNF-α) and M2 marker (IL-10) of lung tissue in different groups (normal, PBS and OASCLR groups) detected using Western blot (WB). (M) Representative immunofluorescence staining images for CD45 (red), IL-10 (red), TNF-α (red) and nucleus (blue). Scale bar, 100 μm. (D and K) Data are presented as mean ± standard deviation (SD). (D) Data are analysed using *t*-test (*n* = 3 biologically independent samples). (K) Data were analysed using two-way ANOVA with multiple comparison test (*n* = 3 biologically independent samples).

To test whether lung microbiome was altered after treatment with OASCLR, mice were subjected to SA to cause primary pneumonia. The experimental outline of primary pneumonia is shown in Fig. 5A. In brief, C57BL/6 mice were infected with SA using a nasal intubation drip on Day –3 and the mice were treated with PBS or OASCLR through non-invasive aerosol inhalation on Day 0. Then blood routine test analysis would be performed on Days 1 and 7, and 16S ribosomal RNA gene sequencing was analysed on Day 2. As shown in Fig. 5B, the levels of white blood cells (WBCs), lymphocytes (lymph#), monocytes (mon#) and granulocytes (gran#) and the percentage of lymphocytes (lymph%) in the PBS group were higher than those of the OASCLR group, suggesting that OASCLR had great treatment effect in primary pneumonia. Emerging evidence has suggested that live probiotics could modulate the gastrointestinal microbiome [50,51] and the intestine would interact with the lung through the gut–lung axis [52]. Thus, this led us to ask whether OASCLR could modulate the lung microbiota in mice. 16S ribosomal RNA gene sequencing could be used to analyse the bacteria diversity at species and strain levels [53]. The lung tissues from the PBS and OASCLR groups were collected to be analysed using 16S ribosomal RNA gene sequencing to obtain data on lung microbiota. The number of operational taxonomic units (OTUs) in the PBS and OASCLR groups were 265 and 789, respectively (Fig. 5C). In addition, the Chao richness index in the OASCLR group was bigger than that in the PBS group (Fig. 5D). Those data indicated that OASCLR treatment significantly improved bacterial richness (observed OTUs richness) and diversity (Chao richness index) in the lung compared with PBS treatment. Further analysis was performed at the phylum level. In contrast to the PBS group, the OASCLR group showed significantly increased relative abundance of Firmicutes and decreased relative abundance of Proteobacteria and Bacteroidota (Fig. 5E). A heat-map diagram was used to display the difference in bacterial species between the PBS and OASCLR groups. Compared with the PBS group, the OASCLR group decreased the content of pathogen bacteria, including Staphylococcus, Acinetobacter, Rodentibacter and Streptococcus, but increased the content of probiotic bacteria, including Blautia, Lactobacillus, Bacillus, Sphingomonas, Ruminococcus and Butyricicoccus, and increased the content of commensal bacteria, including Dorea, Collinsella, Rhodococcus and Bifidobacterium (Fig. 5F). The result revealed that OASCLR could modulate lung microbiota by inhibiting the growth of pathogen bacteria and improving the growth of probiotic bacteria and commensal bacteria. The principal component analysis (PCA) diagram between the PBS and OASCLR groups further showed some differences between the two groups, suggesting that OASCLR treatment could modulate microbiota diversity (Fig. 5G). The partial least squares discriminant analysis (PLS-DA) diagram also displayed some differences between the PBS and OASCLR groups, suggesting some difference in microbial composition between the PBS and OASCLR groups (Fig. 5H). Those data demonstrated that OASCLR could modulate lung microbiota in the lung. It was further analysed whether OASCLR could regulate the overactivated inflammatory response in primary bacterial pneumonia. The status of the immune response of the PBS and OASCLR groups was characterized using ELISA, fluorescence-activated cell sorting (FACS), qRT-PCR, Western blot (WB), immunofluorescence staining and immunohistochemistry. First, the concentration of IL-10 and TNF-α in the serum was detected using ELISA (Supplementary Fig. S22). The ratio between IL-10 and TNF-α in the OASCLR and PBS groups was 0.85 and 0.52, respectively. The ratio between IL-10 and TNF-α in the OASCLR group was bigger than that in the PBS group, indicating that OASCLR treatment could alleviate the overactivated inflammatory response. Then, FACS analysis of the lung tissue of mice in the normal, PBS and OASCLR groups was performed (Fig. 5I)and J). The content of alveolar macrophages (CD11c^high^, CD11b^neg^) in the normal, PBS and OASCLR groups was 25.8%, 75.9% and 52.1%, respectively, and for pro-inflammatory monocytes (CD11b^high^, CD11c^neg^), the normal, PBS and OASCLR groups was 0.5%, 1.11% and 0.65%, respectively (Fig. 5I). These results suggested that the number of pro-inflammatory monocytes and alveolar macrophages in the OASCLR group was decreased compared with that in the PBS group. In addition, lung macrophage (CD45^+^ CD11b^+^) showed that the expression of CD80 (M1 marker) in the OASCLR group was decreased compared with that in the PBS group from 28.7% to 21.4%, indicating M2 polarization (Fig. 5J). Next, RT-PCR was further performed to analyse the gene expression of lung macrophages. Figure 5K)shows that the ratio between TNF-α and IL-10 in the OASCLR group was decreased compared with that in the PBS group. The result revealed that OASCLR treatment could polarize macrophages into M2 phenotype. WB showed a similar result (Fig. 5L)and Supplementary Fig. S23). Then, immunofluorescence analysis of lung tissue showed that the expression of CD45 and TNF-α in the OASCLR group was decreased compared with that in the PBS group, but the expression of IL-10 had slightly increased, demonstrating decreased pro-inflammatory response in lung tissue treated using OASCLR (Fig. 5M). Images of immunohistochemistry staining presented that the expression of TNF-α was decreased and IL-10 was increased in the OASCLR group compared with that in the PBS group (Supplementary Fig. S24). These results demonstrated that OASCLR could regulate the overactivated inflammatory response in primary bacterial pneumonia. Next, toxicity to major organs (heart, liver, spleen, lung and kidney) was detected via hematoxylin and eosin (H&E) staining (Supplementary Fig. S25). As for the PBS group, the lung tissue had inflammatory cells and red blood cells (the inflammatory cells and red blood cells were marked by a green arrow and arrowhead, respectively), suggesting that SA had adverse effects on the lungs of C57BL/6 mice. In contrast, the OASCLR group exhibited no significant tissue damage and adverse effects. In the meantime, toxicity to the liver and kidney was further characterized using blood biochemistry from the mice treated without OASCLR or with OASCLR but without SA treatment. As some indicators of hepatotoxicity and nephrotoxicity, we monitored alkaline phosphatase (AKP), alanine aminotransferase (ALT), blood urea nitrogen (BUN), glucose (GLU), total bilirubin (T-BIL), triglyceride (TG) and white proteins in serum. Compared with healthy mice (untreated mice), the levels of AKP, ALT, BUN, GLU, T-BIL, TG and white proteins in serum from mice treated with OASCLR were almost not altered (Supplementary Fig. S26). The result of H&E staining suggested that OASCLR nanoparticles were generally safe and had biocompatibility in the major organs. The blood biochemistry further confirmed that OASCLR nanoparticles only caused any damage to the liver and kidney. Those data suggested that OASCLR nanoparticles had tremendous therapeutic potential against hyperactive immunocompetent primary pneumonia caused by SA.

### OASCLR enhanced the phagocytosis ability of macrophages in immunocompromised secondary pneumonia

**Figure 6.**
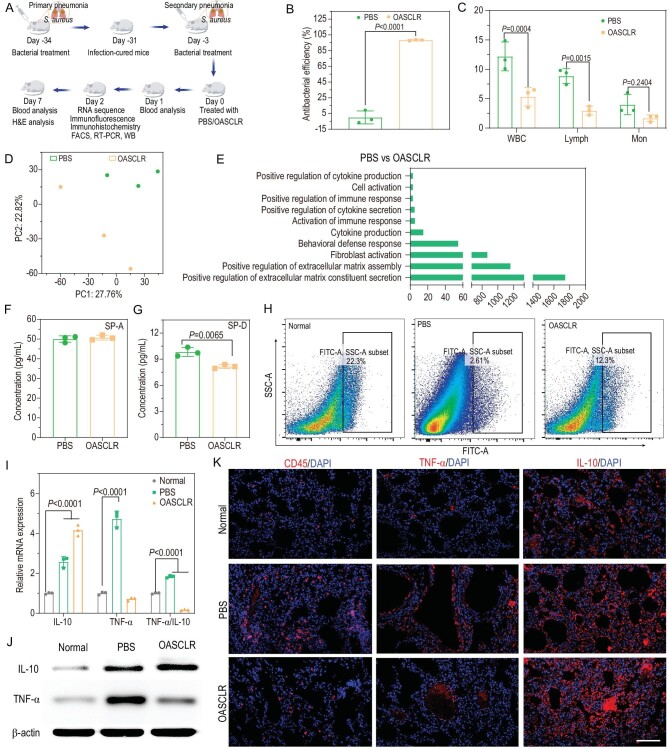
OASCLR enhanced phagocytosis ability of macrophage in immunocompromised secondary pneumonia. (A) Schematic diagram of the experimental outline of secondary pneumonia with SA. (B) The antibacterial efficiency of two groups (PBS and OASCLR). (C) Routine analysis of blood from C57BL/6 for secondary pneumonia. (D) The PCA plot of two groups. (E) The GO functional enrichment analysis of the PBS and OASCLR groups. (F–I) The concentration of (F) SP-A and (G) SP-D of two groups (PBS and OASCLR) analysed using ELISA assay. (H) FACS analysis of lung tissue in mice after intra-tracheal instillation of FITC–*S. aureus*. The normal group represented the mice without any treatments. The PBS and OASCLR groups stood for the mice cured of primary pneumonia and mice in the PBS and OASCLR groups were treated with PBS and OASCLR, respectively. (I) qRT-PCR analysis of gene IL-10 and gene TNF-α expressions in lung tissue of mice. (J) The protein expression of M1 marker (TNF-α) and M2 marker (IL-10) of lung tissue in different groups (normal, PBS and OASCLR groups) detected using Western blot (WB). (K) Representative immunofluorescence staining images for CD45 (red), IL-10 (red), TNF-α (red) and nucleus (blue). Scale bar, 100 μm. Data are presented as mean ± standard deviation (SD). (B, F and G) Data were analysed using t-test (*n* = 3 biologically independent samples). (C and I) Data were analysed using two-way ANOVA with multiple comparison test (*n* = 3 biologically independent samples).

A double-infection model was used to mimic secondary pneumonia to test whether OASCLR nanoparticles could change the state of poor phagocytic ability in macrophages in secondary pneumonia. Mice were first treated with a bacterial (SA) primary pneumonia and then left to recover for 28 days, and after that infected with SA to cause secondary bacterial pneumonia (Fig. 6A). The antibacterial efficiency of the OASCLR nanoparticle *in vivo* is shown in Fig. 6B. Compared with the PBS group, the antibacterial efficiency of the OASCLR group was 98.32 ± 1.10%, suggesting that OASCLR had great antibacterial ability against SA *in vivo*. Blood routine test analysis was further performed to characterize whether OASCLR had treatment efforts in second bacterial pneumonia (Fig. 6C). The values of WBC, lymph# and mon# in OASCLR group were lower than those in the PBS group, suggesting that OASCLR had great treatment effects in secondary pneumonia. The RNA sequencing (RNA-Seq) analysis was further performed to analyse the gene-expression differences of macrophages between two PBS and OASCLR groups. The PCA diagram between the PBS and the OASCLR groups showed some distances between the two groups, suggesting that OASCLR treatment caused gene-expression differences in macrophages compared with the PBS group (Fig. 6D). The result of gene ontology (GO) analysis between the PBS and OASCLR groups showed that the expression of positive regulation of cytokine production, cell activation, positive regulation of immune response, positive regulation of cytokine secretion, activation of the immune response, cytokine production, behavioral defense response, fibroblast activation, positive regulation of extracellular matrix assembly and positive regulation of extracellular matrix constituent secretion in the OASCLR group was upregulated compared with the PBS group (Fig. 6E). The result suggested that OASCLR might regulate the phagocytosis function of macrophages. The signal-regulatory protein α (Sirpa) regulates tyrosine kinase-coupled signaling processes, including phagocytosis [4]. And surfactant proteins SP-A and SP-D are agonists of Sirpa [54]. The concentration of SP-A and SP-D was further measured to assess the macrophage phagocytosis ability (Fig. 6F)and G). Compared with the PBS group, the concentration of SP-A in the OASCLR group was almost unchanged, whereas the concentration of SP-D decreased. The result was correlated with the downregulation of Sirpa expression, suggesting that the expression of Sirpa was inhibited in the lung. Indeed, the inhibition of Sirpa activation demonstrated that the phagocytic activity of macrophages during secondary bacterial pneumonia was improved in these mice after performing OASCLR treatment. FACS analysis showed that the percentage of phagocytosis of FITC–*S. aureus* in the normal, PBS and OASCLR groups was 22.3%, 2.61% and 12.3%, respectively (Fig. 6H). The result demonstrated that the phagocytic activity was partially restored after treatment with OASCLR. Then, it was further characterized whether OASCLR could cause severe inflammatory responses in secondary pneumonia using ELISA, FACS, qRT-PCR, WB, immunofluorescence staining and immunohistochemistry. The concentration of IL-10 and TNF-α in serum was first measured and the ratio between IL-10 and TNF-α in the OASCLR group was bigger than that in the PBS group, suggesting that OASCLR did not elicit a dramatic inflammatory response (Supplementary Fig. S27). In secondary bacterial infection, the phagocytic ability of macrophages was paralysed and the bacterial burden was enhanced. This caused the expression of IL-10 and TNF-α to be upregulated [55]. On the other hand, OASCLR could effectively kill bacteria. The decreased bacterial burden caused the expression of proteins IL-10 and TNF-α to be downregulated [5]. Finally, LR could enhance the phagocytic ability of macrophages and promote the secretion of cytokines IL-10 and TNF-α [56–58] and the expression of cytokine IL-10 was upregulated. FACS analyses indicated that the percentage of alveolar macrophages (CD11c^high^, CD11b^neg^) in the normal, PBS and OASCLR groups was 2%, 9.93% and 3.03%, respectively. The percentage of pro-inflammatory monocytes (CD11b^high^, CD11c^neg^) in the normal, PBS and OASCLR groups was 0%, 16.6% and 4.55%, respectively (Supplementary Fig. S28). The result indicated that OASCLR treatment could decrease the number of alveolar macrophages and pro-inflammatory monocytes. Then, qRT-PCR was further used to assess the phenotype of lung macrophages. Figure 6I)shows that the ratio between TNF-α and IL-10 in the OASCLR group was decreased compared with that in the PBS group, indicating M2-type macrophage polarization. WB showed a similar result (Fig. 6J)and Supplementary Fig. S29). Immunofluorescence staining of the normal, PBS and OASCLR groups was used to analyse the expression of CD45, IL-10 and TNF-α in the lung. The expression of CD45 and TNF-α in the OASCLR group was downregulated compared with that in the PBS group, but the expression of IL-10 had slightly upregulated (Fig. 6K). Immunohistochemistry also showed that the expression of TNF-α was decreased and IL-10 was increased in the OASCLR group compared with the PBS group (Supplementary Fig. S30). The data indicated that OASCLR nanoparticles could effectively clear bacteria, recover phagocytic activity and minimize inflammatory responses to secondary bacterial pneumonia. To further study the biological toxicity of OASCLR to mice in the second bacterial pneumonia model, major organs (heart, liver, spleen, lung and kidney) from the PBS and OASCLR groups were collected for H&E staining (Supplementary Fig. S31). As for the PBS group, the lung tissue had inflammatory cells and fibrin strains (the inflammatory cells and fibrin strains were marked by a red arrowhead and arrow, respectively), suggesting that SA had adverse effects on the lung of C57BL/6 mice. In contrast, the H&E staining slices in the OASCLR group revealed no significant tissue damage and adverse effects, demonstrating that OASCLR had no toxicity to major organs. Finally, the toxicity of OASCLR to kidney and liver was further detected by measuring the levels of AKP, ALT, BUN, GLU, T-BIL, TG and white proteins in serum from the mice treated without OASCLR or with OASCLR but without SA treatment. As shown in Supplementary Fig. S32, the levels of AKP, ALT, BUN, GLU, T-BIL, TG and white proteins in the OASCLR group were not altered compared with untreated group (mice recovered from primary pneumonia). The result indicated that OASCLR had excellent biocompatibility in mice recovering from primary bacterial pneumonia. The result suggested that OASCLR had tremendous therapeutic effect against immunocompromised secondary pneumonia caused by SA.

## CONCLUSION

In summary, the OASCLR nanoparticle was centered on restoring host immunity rather than avoiding bacterial colonization. The OASCLR nanoparticle could regulate the pro-inflammatory state and modulate the balance of the lung microbiome in primary bacterial pneumonia. Meanwhile, OASCLR nanoparticles could also enhance the macrophage's phagocytic ability to improve the innate immune response to secondary bacterial pneumonia. Our strategy might also provide a promising platform for treating diseases other than bacterial pneumonia. The OASCLR nanoparticle could evade immune attack through a CS/HA–ononin shell and be effectively clear after the loss of encapsulation, guaranteeing their clinical translation practically. The combination of probiotics and biomaterial not only can boost the function of probiotics but also can make up for the lack of biological materials. The OASCLR nanoparticle has the potential for treating various diseases other than bacterial pneumonia.

## MATERIALS AND METHODS

### Synthesis of SCLR, OSCLR, ASCLR, OASCLR, OASCLR Cy 5.5, OSCLR Cy 5.5 and FITC–OASCLR

CS solution (10 mg/mL) was prepared in 1% (v/v) glacial acetic acid solution. HA solution (20 mg/mL) was dissolved in deionized water and ononin (1 mg/mL) was dispersed in PBS solution. The LR pellet was isolated from the broth solution (4 mL) after centrifugation (6000 rpm, 5 min). Then, LR pellet was resuspended in the solution (PBS (3 mL) and CS solution (1 mL)) and the mixture was thoroughly vortexed to create a homogenous solution and incubated on a shaker for 30 min. Then the homogenous solution was centrifuged and the bacterial pellet collected. The SCLR nanoparticles were obtained after the bacterial pellet had been washed with PBS. If the solution was composed of PBS (3 mL), CS solution (1 mL), ononin (80 μL) and Cy 5.5 (2 μL, 0.1 μM), then the formed nanoparticles were named as OSCLR Cy 5.5 nanoparticles. Next, HA was further used to coat SCLR to form another layer, which was named ASCLR. On the other hand, if HA (1 mL) was mixed with ononin (80 μL) or FITC–ononin (80 μL), then the formed nanoparticles were named as OASCLR nanoparticles or FITC–OASCLR nanoparticles. If the mixture (HA (1 mL), ononin (80 μL) and Cy 5.5 (2 μL, 0.1 μM) was used to coat SCLR, then the formed nanoparticles were named as OASCLR Cy 5.5.

### Resistance assessment *in vitro*

LR (10^8^ CFU/mL), SCLR (10^8^ CFU/mL) and OASCLR (10^8^ CFU/mL) were resuspended into 1-mL solutions (50% ethanol (v/v), NaOH (pH = 13), HCl (pH = 1) and penicillin/streptomycin (penicillin (10 000 U/mL)–streptomycin (10 mg/mL))) for 2 h, respectively. Then, the bacteria cells were fixed in 2.5% glutaraldehyde for 4 h. Next, the bacterial cells were dehydrated in 10%, 30%, 50%, 75%, 90% and 100% ethanol (v/v) for 15 min. The images were obtained using SEM.

### Statistical analyses

Data were presented as mean ± standard deviation (SD). The paired or unpaired *t*-test, one-way analysis of variance (ANOVA) and two-way ANOVA were used in statistical analyses. *P* < 0.05 was considered statistically significant.

## Supplementary Material

nwac221_Supplemental_File
