# Supplementary material for: Probiotic-based nanoparticles for targeted microbiota modulation and immune restoration in bacterial pneumonia
Source: Natl Sci Rev. 2022 Oct 16;10(2):nwac221. doi: 10.1093/nsr/nwac221 (PMC9935993; doi:10.1093/nsr/nwac221)
Supplement: nwac221_Supplemental_File [file nwac221_supplemental_file.docx]

Supplementary Material

**Probiotic-based nanoparticle for targeted microbiota modulation and immune restoration in bacterial pneumonia**

Jieni Fu^1,2^, Xiangmei Liu^3^, Zhenduo Cui^1^, Yufeng Zheng^2^, Hui Jiang^1^, Yu Zhang^4^, Zhaoyang Li^1^, Yanqin Liang^1^, Shengli Zhu^1^, Paul K Chu^5^, Kelvin Wai Kwok Yeung^6^, Shuilin Wu^1,2^*

The file includes:

**Material and methods**

**Supplementary Fig. 1.** TEM images of uncoated LR, SCLR, and OASCLR. Scale bar, 1 μm.

**Supplementary Fig. 2.** The images of bacterial live/dead staining of LR, SCLR, and OASCLR. SYTO 9 and PI were used to stain live and dead bacteria, and live and dead bacteria exhibited green and red fluorescence, respectively. Scale bar, 10 μm.

**Supplementary Fig. 3.** The images of LR and FITC-OASCLR. LR was fixed by 4 % formaldehyde and stained by PI. Scale bar, 10 μm.

**Supplementary Fig. 4.** Method for calculating the loading amount of CS, HA, and ononin loaded on the surface of LR. UV-Vis absorbance spectra of difference concentrations of (A) CS and (B) OA (a mixture of HA and ononin). (C) CS concentration-OD value (285 nm) curves and linear regression analysis. (D) OA concentration-OD value (285 nm) curves and linear regression analysis. (E) UV-Vis absorbance spectra of remaining solutions during the synthesis of SCLR and OASCLR after diluting twice with PBS.

**Supplementary Fig. 5.** The SEM images of LR, SCLR, and OASCLR cultured in different environment (ethanol (50%, v/v), NaOH (pH=13), HCl (pH=1), and penicillin/streptomycin (10000 U/mL)) at 37 °C for 2 h. Scale bar, 0.5 μm.

**Supplementary Fig. 6.** The SEM images of LR, SCLR, and OASCLR cultured in simulated pulmonary environment pH (pH=6) at 37 °C for 2 h. Scale bar, 0.5 μm.

**Supplementary Fig. 7.** The images of bacterial live/dead staining of LR, SCLR, and OASCLR after culturing with different environment (ethanol (50%, v/v), NaOH (pH=13), HCl (pH=1), penicillin/streptomycin (10000 U/mL), and simulated pulmonary environment pH (pH=6)) at 37 °C for 2 h. SYTO 9 and propidium iodide (PI) were used to stain live and dead bacteria into green and red color, respectively. Scale bar, 10 μm.

**Supplementary Fig. 8.** Flow cytometry results of CD206 (M2 marker) and CD80 (M1 marker) expression after 24 h treatment with medium only, LPS (100 ng/mL), and IL-4 (20 ng/mL).

**Supplementary Fig. 9.** (**A**) Left: Confocal microscopy image of macrophages pre-treated for 24 h with culture medium, followed by 1 h incubation with OASCLR-Cy 5.5 (10^7^ CFU/mL). Right: Confocal microscopy image of macrophages pre-treated for 24 h with IL-4 (20 ng/mL), followed by 1 h incubation with OASCLR-Cy 5.5 (10^7^ CFU/mL). Hoechst 33342 was used for nuclei staining. Scale bar, 10 μm. (**B**) Left: The confocal microscopy image of macrophages pre-treated for 24 h with LPS (100 ng/mL), incubated with anti-CD44 antibody (500 μg/mL), followed by treatment with OASCLR-Cy 5.5 (10^7^ CFU/mL) for 1 h. Right: The confocal microscopy image of macrophages pre-treated for 24 h with LPS (100 ng/mL), followed by treatment with OSCLR-Cy 5.5 (10^7^ CFU/mL) for 1 h. OSCLR was OASCLR without HA. Hoechst 33342 was used for nuclei staining. Scale bar, 10 μm.

**Supplementary Fig. 10.** (A) The spread plate pictures of LR after incubating with different concentrations of ononin (0 μg/mL, 2.5 μg/mL, 5 μg/mL, 10 μg/mL, 20 μg/mL, and 40 μg/mL) for 24 h. (B) The CFU value of LR after incubating with different concentrations of ononin (0 μg/mL, 2.5 μg/mL, 5 μg/mL, 10 μg/mL, 20 μg/mL, and 40 μg/mL). Data are presented as mean ± standard deviation (SD). Data was analyzed by one-way ANOVA with multiple comparison test. (n = 3 biologically independent samples).

**Supplementary Fig. 11.** (A) The spread plate pictures of PV, EC, and MREC after treating for 24 h with different concentrations of ononin (0 mg/mL, 0.25 mg/mL, 0.5 mg/mL, 1 mg/mL, 2 mg/mL, and 4 mg/mL). The CFU value of (B) PV, (C) EC, and (D) MREC after treating for 24 h with different concentrations of ononin (0 mg/mL, 0.25 mg/mL, 0.5 mg/mL, 1 mg/mL, 2 mg/mL, and 4 mg/mL). B-D. Data are presented as mean ± standard deviation (SD). Data was analyzed by one-way ANOVA with multiple comparison test. (n = 3 biologically independent samples).

**Supplementary Fig. 12.** The spread plate pictures of PV and OASCLR after incubating PV with different concentration of OASCLR for 24 h.

**Supplementary Fig. 13.** The spread plate pictures of *Salmonella typhimurium* (ST) and OASCLR after incubating ST with different concentration of OASCLR for 24 h.

**Supplementary Fig.14.** The spread plat pictures of MRSA and OASCLR after incubating MRSA with different concentrations of OASCLR for 24 h.

**Supplementary Fig.15.** The spread plat pictures of MREC and OASCLR after incubating MREC with different concentrations of OASCLR for 24 h.

**Supplementary Fig.16.** The spread plat pictures of EC and OASCLR after incubating EC with different concentrations of OASCLR for 24 h.

**Supplementary Fig.17.** The spread plat pictures of SA and OASCLR after incubating SA with different concentrations of OASCLR for 24 h.

**Supplementary Fig. 18.** The growth condition of bacteria, and the initial bacterial number of OASCLR was 10^7^ CFU/mL. Data are presented as mean ± standard deviation (SD). Data was analyzed by one-way ANOVA with multiple comparison test. (n = 3 biologically independent samples).

**Supplementary Fig.19.** The spread plat pictures of SA_P1_ and OASCLR after incubating SAP1 with different concentrations of OASCLR for 24 h.

**Supplementary Fig. 20.** The spread plat pictures of SA_P2_ and OASCLR after incubating SA_P2_ with different concentration of OASCLR for 24 h.

**Supplementary Fig. 21.** The spread plat pictures of SA_P3_ and SA_P0_ after treating with different CM.

**Supplementary Fig. 22.** The concentration of protein IL-10 and protein TNF-α of two groups (PBS and OASCLR) analyzed by ELISA assay. Data are presented as mean ± standard deviation (SD). Data was analysed by t-test. (n = 3 biologically independent samples).

**Supplementary Fig. 23.** qPCR analysis of gene IL-10 and gene TNF-α expressions in lung tissue of mice. Data are presented as mean ± standard deviation (SD). Data was analysed by two way ANOVA. (n = 3 biologically independent samples).

**Supplementary Fig. 24.** Immunohistochemical staining of IL-10 and TNF-α in the mice lung tissue. Scale bar, 50 μm.

**Supplementary Fig. 25.** Histological examinations of H&E-stained major organ sections, including heart, liver, spleen, lung, and kidney. The inflammatory cells and red blood cells were marked by green arrow and arrowhead, respectively. Scale bar, 20 μm.

**Supplementary Fig. 26.** Evaluation of the hepatotoxicity and nephrotoxicity of untreated and OASCLR groups (according to the serum levels of AKP, ALT, BUN, GLU, T-BIL, TG, and white proteins) after different treatments. (n = 6 biologically independent samples).

**Supplementary Fig. 27.** The concentration of IL-10, and TNF-α of two groups (PBS and OASCLR) analyzed by ELISA assay. Data are presented as mean ± standard deviation (SD). Data was analyzed by t test. (n = 3 biologically independent samples).

**Supplementary Fig. 28.** Characterization of pro-inflammatory monocytes/alveolar macrophages in lung tissue of Normal, PBS, and OASCLR groups. Subsets (gated on CD45^+^LY6G^-^) were characterized based on CD11b and CD11c expression as pro-inflammatory monocytes (CD11b^high^, CD11c^neg^) and alveolar macrophages (CD11c^high^, CD11b^neg^).

**Supplementary Fig. 29.** qPCR analysis of gene IL-10 and gene TNF-α expressions in lung tissue of mice. Data are presented as mean ± standard deviation (SD). Data was analyzed by two way ANOVA. (n = 3 biologically independent samples).

**Supplementary Fig. 30.** Immunohistochemical staining of IL-10 and TNF-α in the mice lung tissue. Scale bar, 50 μm.

**Supplementary Fig. 31.** Images of H&E staining for major organs, including heart, liver, spleen, lung, and kidney. (The inflammatory cells and fibrin strains were marked by red arrowhead and arrow, respectively.) Scale bar, 20 μm.

**Supplementary Fig. 32.** Evaluation of the hepatotoxicity and nephrotoxicity of untreated and OASCLR groups (according to the serum levels of ALT, BCA, BUN, GLU, TG, and white proteins) after different treatments. (n = 6 biologically independent samples).

**Supplementary Table 1.** Antimicrobial activity of LR and OASCLR against PV, ST, MRSA, MREC, EC, and SA. MBC* is defined as the concentration of sample, which could lead the value of CFU to becoming zero. The concentration of LR and OASCLR was chosen as MBC when the spread plate appeared several bacteria.

**Materials and methods**

**Materials and strains.** Chitosan (CS) and hyaluronic acid (HA) were pursued from Heowns Technology Co., Ltd. (Tianjin, China). Glacial acetic acid was from Chemart Chemical Technology Co., Ltd. (Tianjin, China). Ononin was from Push Bio-technology. Trypsin-EDTA, penicillin and streptomycin, FITC-conjugated phalloidin, and 4', 6-diamidino-2-phenylindole (DAPI) were from yeasen. Fetal bovine serum (FBS) was from Gibco. PrimeScript RT Master Mix and 2×SYBR Premix Ex Taq II were from TaKaRa. 3-[4,5-dimethylthiazol-2-yl]-2,5-diphenyl tetrazolium bromide (MTT) assay kit and LDH cytotoxicity assay kit were purchased from beyotime. Lactic acid assay kit and hydrogen peroxide assay kit were from Nanjing Jiancheng Bioengineering Institute. AKP assay, ALT assay, BUN assay, GLU assay, T-BIL assay, TG assay, and white proteins assay were from Nanjing Jiancheng Bioengineering Institute. MRS broth were pursued from Solarbio life sciences.

*Staphylococcus aureus* (SA; American Type Culture Collection (ATCC) 25923), *Escherichia coli* (EC; ATCC 8099), Gram-positive methicillin-resistant *S. aureus* (MRSA; China Center for type culture collection (CCTCC) 16465), multi-resistant *E. coli* (MREC; China Center of Industrial Culture Collection (CICC) 10663)*,* *Lactobacillus rhamnosus* (LR; CCTCC AB 2015377), *Proteusbacillus vulgaris* (PV; CCTCC AB 91103), and *Salmonella typhimurium* (ST; CCTCC PB 2019001) were purchased from China General Microbiological Culture Collection Center (CGMCC).

**Synthesis of FITC-ononin.** Ononin (8 mg) was dispersed in 2 mL water, and then adjusted pH to 8.5 with 0.5 M NaHCO_3_. Next, FITC (4 mg) was added to the above solution and reacted overnight with magnetic stirring. Then, ethanol was added to the above solution, and the final concentration of ethanol was 80% (v/v). Finally, FITC-ononin was obtained by centrifugation and washed with water three times.

**Characterization of LR, SCLR, and OASCLR.** The morphological images of LR, SCLR, and OASCLR were obtained by SEM (JSM-7800F, Japan; Sigma 300, Germany; Apreo S LoVac, USA). TEM (JEOL-2100F, Japan) was used to visualize the morphologies of LR, SCLR, and OASCLR. The chemical structure of ononin was measured through FTIR (Nicolet IS10, United States).

**Cells culturing.** The RAW 264.7 macrophage cells, MC3T3-E1 osteoblast cells, L929 fibroblast cells, Caco-2 epithelial cells, and A549 epithelial cells were from Nankai University. These cells were cultured in a growth medium at 37 °C in 5% CO_2_ atmosphere. The growth medium contained basic medium, FBS, and penicillin (10, 000 U/mL)-streptomycin (10 mg/mL) liquid at a volume ratio of 89: 10: 1. As for RAW 264.7, the basic medium was Roswell park memorial institute (RPMI) 1640 (1×). And as for Caco-2, L929, and MC3T3-E1, the basic medium was Dulbecco's Modified Eagle Medium (DMEM). And the basic medium of A549 was Dulbecco's Modified Eagle Medium/Nutrient Mixture F-12 (DMEM/F-12) (1:1).

SA, EC, MRSA, MREC, PV, and ST were grown at 37 °C in lysogeny broth (LB) medium (1% tryptone (w/v), 0.5% yeast extract (w/v) and 1% NaCl (w/v)). LR was grown at 37 °C in MRS broth.

**Calculation of loading** **amount of** **CS, HA, and ononin on OASCLR.** The amount of CS, HA, and ononin loaded on OASCLR is defined as the mass by subtracting the remaining material in the solution from the initial material added, and the remaining material in the solution was characterized by UV-vis Spectrophotometer (UV-2700, China). First, LR (4×10^9^ CFU/mL, 1 mL) and CS (10 mg/mL, 1 mL) were mixed with 2 mL PBS solution and shaken on a shaker at 37 ℃ for 30 min. Then the SCLR nanoparticles were obtained by centrifugation (6000 rpm, 5 min), and the remaining solution was collected for following calculating the amount of CS. Second, the SCLR nanoparticles were washed with PBS solution two times. And then, the washed SCLR nanoparticles were re-dispersed in 1 mL PBS solution. The HA (20 mg/mL, 1 mL) and ononin (1 mg/mL, 80 μL) were evenly mixed to form a mixture. Next, the mixture (1 mL), PBS solution (2 mL), and SCLR (10^9^ CFU/mL, 1 mL) were mixed and shaken on a shaker at 37 ℃ for 30 min. Finally, then the OASCLR nanoparticles were obtained by centrifugation (6000 rpm, 5 min), and the remaining solution was collected for following calculating the amount of HA and ononin.

**Bacterial fluorescence assays.** The interaction between ononin and LR: LR pellet was isolated from the broth solution (1 mL) after centrifugation (6000 rpm, 5 min) and washed with PBS three times. Then, the LR was fixed with 4% formaldehyde for 20 min and stained with PI (1.67 mM, 0.1 μL/100 mL). Next, LR labeled with PI was used to synthesize FITC-OASCLR. Finally, the images were obtained by confocal microscopy (Nikon A1R+, Japan).

Bacterial live/dead staining: The bacterial viability was characterized by a LIVE/DEAD® BacLight^TM^ Bacterial Viability Kits (Introgen). LR, SCLR, and OASCLR were performed different treatments for 2 h, such as ethanol (50%, v/v), NaOH (pH=13), HCl (pH=1), penicillin/ streptomycin (10000 U/mL), and simulated pulmonary environment pH (pH=6). Then, the bacteria were stained with a mixture of PI (1.67 mM, 0.1 μL/100 mL) and SYTO 9 (1.67 mM, 0.1 μL/100 mL). Finally, the images were obtained by confocal microscopy (Nikon A1R+, Japan).

**Assessment of the breakdown products of hyaluronan.** The detailed steps could be seen in previous literature [1]. HA could break into N-acetyl-D-glucosamine after exposure to hyaluronidase (HYAL). Briefly, OASCLR (10^9^ CFU) and HA (1 mg/mL) were treated with 100 IU/mL of HYAL at 37 °C for 6 h. Then, taking out 100 μL samples mixed with 400 μL PBS and heated for 5 min at 100 °C. Next, 0.1 mL potassium tetraborate (0.8 M, pH=9.0) was added to the mixture, and they were further heated for 5 min at 100 °C. And then, 3 mL *p*-dimethylaminobenzaldehyde (DMAB) (1 g DMAB dissolved in 10 mL glacial acetic acid containing 12.5% (v/v) of 10 M HCl) was added into the mixture and cultured at 37 °C for 20 min. Finally, the solution was measured at 544 nm using a microplate reader.

**ROS-responsiveness of OASCLR.** First, 50 μM dichlorofluorescin diacetate (DCFDA) was incubated with AAPH (1 mM) in the presence of OASCLR or PBS for 1 h at 37 °C. The data was obtained through a microplate reader at 490 nm excitation and 520 nm emission.

**LDH Cytotoxicity Assay.** The RAW 264.7, A549, Caco, L929, and MC3T3-E1 cell lines were seeded in a 96-well plate for 24 h at 37 °C. Then, removed the medium and added different samples (fresh medium, LR (10^9^ CFU/mL), ASCLR (10^9^ CFU/mL), and OASCLR (10^9^ CFU/mL)). Next, the plates were incubated for 1 h, 2 h, 3 h, and 24 h. At appropriate time points, the cells were treated by LDH cytotoxicity assay. Finally, the data was obtained by microreader at 490 nm.

***In vivo* and *in vitro* ELISA analysis.** The concentrations of cytokines in cell growth medium and blood serum were measured by ELISA assay kit (Shanghai Enzyme-linked Biotechnology Co., Ltd.).

**RT-qPCR analysis.** RAW 264.7 cells (10^4^ cells/well) were grown at a 12-well plate and incubated for 24 h at 37 °C. Then, removed the growth medium and added different mediums (fresh medium, fresh medium containing LR (10^7^ CFU/mL), and fresh medium with OASCLR (10^7^ CFU/mL)). After culturing for 48 h, RNA was extracted through total RNA Kit I (Omega Bio-tek). Reverse transcription and quantitative PCR were performed with TB Green PrimeScript RT Master Mix (Takara Bio) and TB Green Premix Ex Taq II (Takara Bio), respectively. TNF-α (Forward: 5'-GACGTGGAACTG GCAGAAGAG-3'; Reverse: 5'TTGGTGGTTTGTGAGTGTGAG-3'), IL-10 (Forward: 5'-GCTC TTACTGACTGGCATGAG-3'; Reverse: 5'-CGCAGCTCTAGGAGC ATGTG-3'). GAPDH (Forward: 5'-TCTCTGCTCCTCCCTGTTCT-3'; Reverse: 5'-CTTGCC GTGGGTAGAGTCAT-3').

**Alamar blue assay.** The 200 μL LR, SCLR, and OASCLR (10^7^ CFU/mL) were added to a 96-well plate and incubated for 24 h at 37 °C. Bacteria proliferation activity was obtained through Alamar blue cell viability assay reagent (Beijing Solarbio Science & Technology Co., Ltd). The data were obtained at 530 nm excitation and 590 nm emission.

***In vitro* bacterial competition.** Various OASCLR (10^9^ CFUs, 10^7^ CFUs, and 10^5^ CFUs) were mixed with pathogens (SA, EC, MRSA, MREC, PV, and ST), respectively. And the bacterial cells were diluted 10^7^ CFU/mL with MRS broth. The mixtures were incubated in MRS broth at 37 °C for 24 h. After co-culturing, the suspension was diluted to a suitable multiple. Next, a solid LB plate was used to spread it and incubated at 37 °C for 24 h for bacterial counting.

As for experiments on bacterial resistance, different passages of OASCLR and SA were used. In the first inhibitory-growth cycle, SA was washed with PBS and collected by centrifugation at 6000 r for 5 min. Next, SA was resuspended in MRS broth. 2 μL SA (10^9^ CFU/mL) was added in 200 μL OASCLR (10^7^ CFU/mL) at 96-well plate and then cocultured with SA for 24 h. The survival bacteria (LR and SA) were collected after the first inhibitory-growth cycle. The SA was grown on selective BBL mannitol salt agar, and LR was grown on MRS agar. The SA after the above steps was named SA_P1_. And LR were performed a complete preparation process to form OASCLR, which was called OASCLR_P1_. The obtained bacteria were further performed the second inhibitory-growth cycle. The detail was the same as in the first inhibitory-growth cycle. These experiments were performed in the atmospheric environment.

**Measurement of MBC.** A series of LR and OASCLR was prepared by gradient dilution, and the final volume was 100 μL in each well (96-well plate). The concentration of LR contained 8 × 10^9^ CFU/mL, 4 × 10^9^ CFU/mL, 2 × 10^9^ CFU/mL, 1 × 10^9^ CFU/mL,5 × 10^8^ CFU/mL, 2.5 × 10^8^ CFU/mL, 12.5 × 10^8^ CFU/mL, 6.25 × 10^8^ CFU/mL, 3.13 × 10^8^ CFU/mL, 1.56 × 10^8^ CFU/mL, 7.81 × 10^7^ CFU/mL and 3.90 × 10^7^ CFU/mL. And The concentration of LR contained 8 × 10^9^ CFU/mL, 4 × 10^9^ CFU/mL, 2 × 10^9^ CFU/mL, 1 × 10^9^ CFU/mL,5 × 10^8^ CFU/mL, 2.5 × 10^8^ CFU/mL. Bacteria (SA, EC, MRSA, MREC, PV, and ST) were diluted into 2× 10^7^ CFU/mL with MRS broth, and each well of a 96-well plate was added 100 μL bacteria solution. The 96-well plate was placed on a shaker at 37 ℃ for 24 h. Each bacterial solution was directly spread on an agar plate and incubated at 37 ℃ for 24 h. MBC is considered as the minimum agent concentration that causes bacterial death.

***In vitro*** **OASCLR targeting assay.** First, the cells were seeded in 24-well plates (10^4^ per well) and incubated for 24 h. IL-4 (20 ng/mL), lipopolysacchride (LPS, 100 ng/mL), growth medium were used to polarize macrophages into M2-type macrophage, M1-type macrophage, and M0-type macrophages, respectively. After 24 h, OASCLR Cy 5.5 (10^7^ CFU/mL) was used to treated different cells. Hoechst 33342 was used to stain cell nucleus. For completion of the study, on the one hand, OSCLR Cy 5.5 (10^7^ CFU/mL, free HA) was used to treat M1-type macrophages. On the other hand, M1-type macrophages were pre-treated for 1 h with anti-CD44 antibody (500 μg/mL) and then cultured with OASCLR Cy 5.5. Finally, the images were obtained with confocal microscopy (Nikon A1R+, Japan).

**Fluorescent staining of cells.** First, each well (12-well plate) was seeded 10^5^ A549 cells. After culturing for 24 h, the growth medium was removed, and added new medium (F12K with 2% trypsin treated by TPCK) with H1N1 (10^7^ plaque forming units (PFU)/mL; 0.1% v/v). Next, the cells treated with H1N1 were incubated for 2 h. And then, the above medium was removed and added fresh growth medium (98% DMEM/F-12 and 2% FBS) containing 10% PBS or 10% oseltamivir phosphate (100 mg/mL), or 10% OASCLR (10^7^ CFU/mL), respectively. After culturing for 24 h, the fresh medium was removed and fixed with 4% paraformaldehyde. Then, the cell cytoskeleton and cell nucleus were stained with phalloidin-TRITC and DAPI, respectively.

As for RAW 264.7, 10^4^ cells were seeded in a 24-well plate. After culturing for 24 h, the growth medium (90% RPMI 1640 and 10% FBS) was removed and added fresh medium containing 10% PBS or 10% OASCLR (10^7^ CFU/mL), respectively. And then, the medium was removed after 24 h, and those samples were fixed with 4% paraformaldehyde. Then, the cell cytoskeleton and cell nucleus were stained with phalloidin-FITC and DAPI, respectively [2].

**Animals.** 72 mice (20 g, C57BL/6J) were used in two models, and each model had 36 mice. All animal experiments were approved by Animal Ethical and Welfare Committee (AEWC) of the Institute of Radiation Medicine, Chinese Academy of Medical Sciences (Approval No. YSY-DWLL-2021016). And all animals were cohoused for four days. Next, all mice inhaled 50 μL SA (10^9^ CFUs) and cohoused for three days.

**The First Animal Model.** The 36 mice were divided into three groups, and each group had 12 mice. The three groups were named as normal, PBS, and OASCLR groups. Normal group was healthy mice without treatment. PBS group was treated with nebulized PBS. And the OASCLR group was treated by nebulized OASCLR in MRS broth. Finally, the mice were performed biosafety analysis, microbiome analysis, qRT-PCR of lung tissue, WB of lung tissue, immunofluorescence staining of lung tissue and immunohistochemical staining of frozen sections of lung tissue at right point in time.

**Biosafety analysis.** Major organs (heart, liver, spleen, lung, and kidney) form two groups were collected to performed H&E staining. Meanwhile, the two groups were performed a routine analysis of blood on day 1 and day 7. The hepatotoxicity and nephrotoxicity of the two groups were evaluated on day 2.

**Microbiome analysis.** Different lung tissues (PBS and OASCLR groups) were collected for 16 S analysis. Briefly, DNA was collected through TruSeqTM DNA Sample Prep Kit. Extracted DNA was analyzed *via* the MiSeq Illumina sequencing platform.

**The second Animal Model.** Through intraperitoneal injection, the 36 mice were treated with gentamicin (30 mg/kg). The mice were cohoused for 28 days after the cure.

And then, the 36 mice inhaled 50 μL SA (10^9^ CFUs) and cohoused for three days. Next, those mice were divided into three groups, and each group had 12 mice. The groups were named as normal, PBS group and OASCLR group, respectively. Normal group was healthy mice without treatment. PBS group was treated by nebulized PBS. The OASCLR group was treated by nebulized OASCLR in MRS broth. Finally, the mice were performed biosafety analysis, RNA-sequence analysis of lung tissue, qRT-PCR of lung tissue, WB of lung tissue, immunofluorescence staining of lung tissue and immunohistochemical staining of frozen sections of lung tissue at right point in time.

**Biosafety analysis.** For histological analysis, H&E staining was performed in major organs (heart, liver, spleen, lung, and kidney) from two groups. Meanwhile, the two groups were performed a routine analysis of blood.

***In vivo* antibacterial assay.** The second animal model was used to assess *in vivo* antibacterial experiments. The lung tissue was ground and suspended in PBS. Next, the suspended solution was spread in solid LB plates. Finally, the solid LB plates were cultured for 24 h at 37 °C.

**RNA-sequence Analysis.** Extra total RNA was extracted by TRIzol reagent (Invitrogen). The concentration and purity of RNA was assessed by Nanodrop 2000 and agarose gel electrophoresis, respectively. The Majorbio Cloud Platform was used to analyze the data. RSEM (version 1.3.1) was used to evaluate the relationship between samples. DESeq2, DEGseq, and edgeR were used to obtain gene differential expression analysis. And gene differential expression analysis was further applied at gene ontology (GO). And GO analysis was processed using Fisher's exact test and the *χ*^2^-test.

***In vivo* flow cytometry.** The lung tissues from different groups were immersed in ice-cold PBS, and then minced with a razor blade. Next, DMEM containing Collagenase A (2.0 mg/mL) and DNase I (50 units/mL) was used to incubate above tissue. Then, 70 μm nylon strainers were chosen to filter the above tissue to obtain single cell suspensions. Next, the cells were incubated with Fc Receptor Binding Inhibition to exclude dead cells. And then, cells were incubated in PBS containing EDTA (1.0 mM) and 5% FBS along with suggested fluorescently labeled primary monoclonal antibodies. The antibodies were anti-CD11c-PE-Cy7, anti-CD80-PE, anti-CD 11b-FITC, anti-CD206-Alexa Fluor 647, anti-CD45-APC-Cy7, anti-LY6G-percp-Cy5.5. Finally, data were obtained by FACSCalibur (BD-Biosciences, New Jersey, USA).


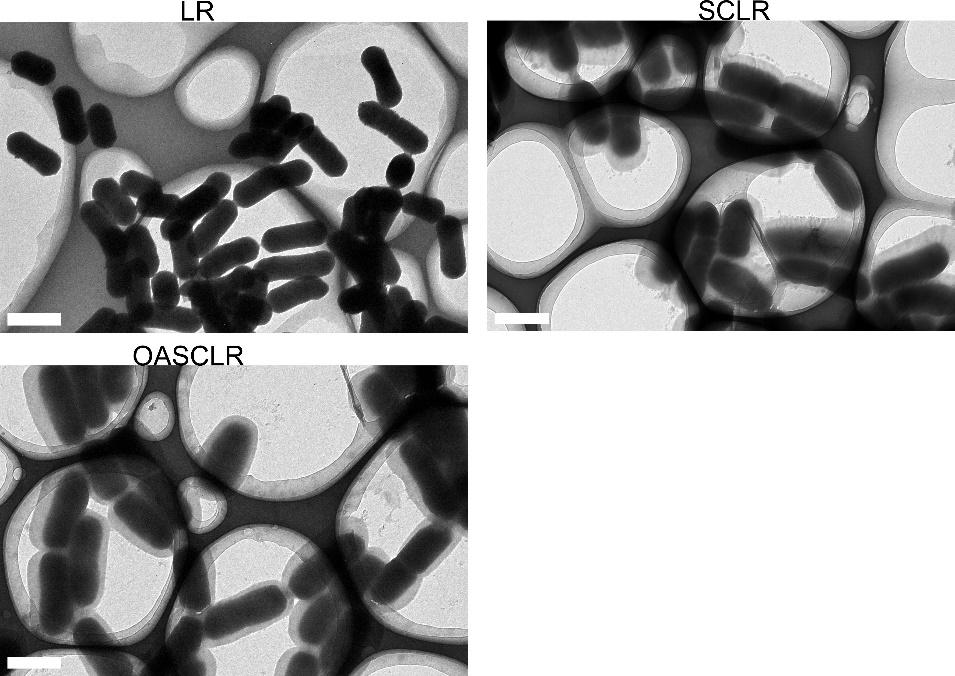


**Supplementary Fig. 1.** TEM images of uncoated LR, SCLR, and OASCLR. Scale bar, 1 μm.


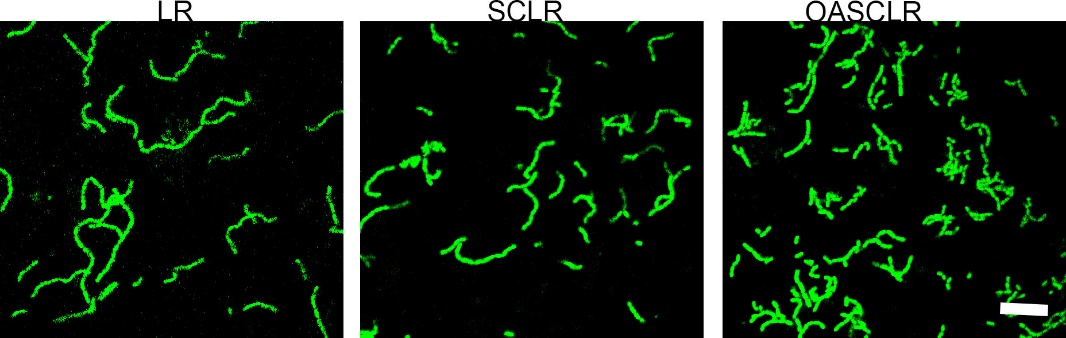


**Supplementary Fig. 2.** The images of bacterial live/dead staining of LR, SCLR, and OASCLR. SYTO 9 and PI were used to stain live and dead bacteria, and live and dead bacteria exhibited green and red fluorescence, respectively. Scale bar, 10 μm.


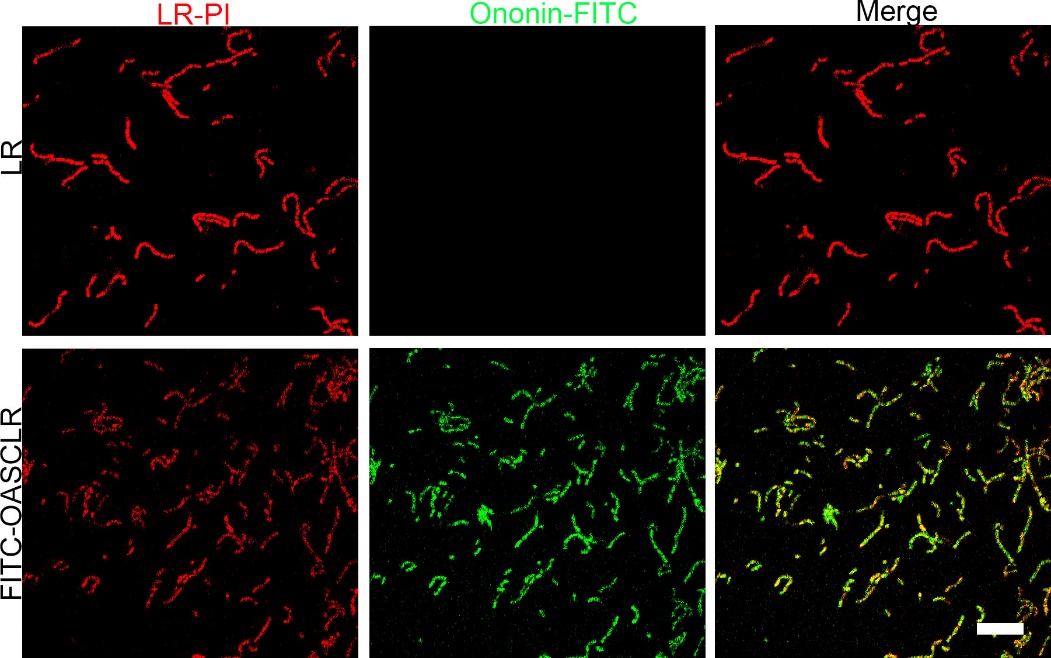


**Supplementary Fig. 3.** The images of LR and FITC-OASCLR. LR was fixed by 4 % formaldehyde and stained by PI. Scale bar, 10 μm.


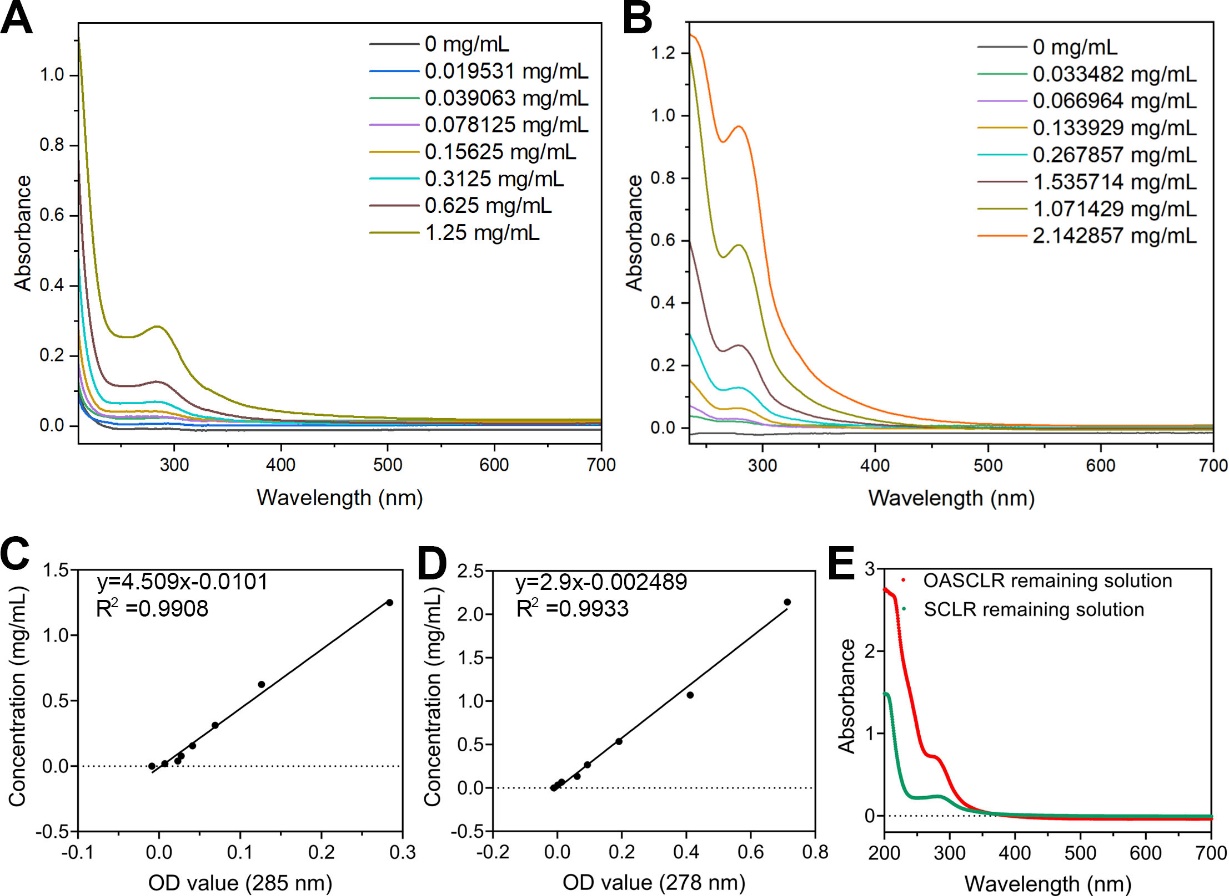


**Supplementary Fig. 4. Method for calculating the** **amount of CS, HA, and ononin loaded on OASCLR.** UV-Vis absorbance spectra of difference concentrations of (**A**) CS and (**B**) OA (a mixture of HA and ononin). (**C**) CS concentration-OD value (285 nm) curves and linear regression analysis. (**D**) OA concentration-OD value (285 nm) curves and linear regression analysis. (**E**) UV-Vis absorbance spectra of remaining solutions during the synthesis of SCLR and OASCLR after diluting twice with PBS.

**Method used for calculating amount of CS, HA, and ononin loaded on the surface of LR:**

As shown in **Supplementary Fig. 4A, 4B**, the UV-Vis spectra of CS and OA indicated that the largest absorption peak appeared at 285 nm and 278 nm, respectively. Then, collected the data and performed linear regression analysis (**Supplementary Fig. 4C, 4D**). Next, **Supplementary Fig. 4E** showed that absorbed value for SCLR remaining and OASCLR remaining solutions was 0.234 and 0.713, respectively. The calculation is as following: the amount CS in solution was 2.09 mg/mL ((4.509×0.234-0.0101) × 2). The amount of CS loaded on OASCLR was 0.41 mg/mL (2.5-2.090012). In the same way, the amount of HA and ononin loaded on OASCLR was 0.16 mg/mL (4.285714 - (0.713×2.9-0.002489) × 2) and 0.31 μg/mL (8.571428 - (0.713×2.9-0.002489) × 2). Finally, the amount of CS, HA, and ononin on OASCLR was 0.41 mg CS, 0.16 mg HA, and 0.31 μg ononin when the LR was 10^9^ CFU. And the amount of CS on OASCLR was 0.41 mg CS.


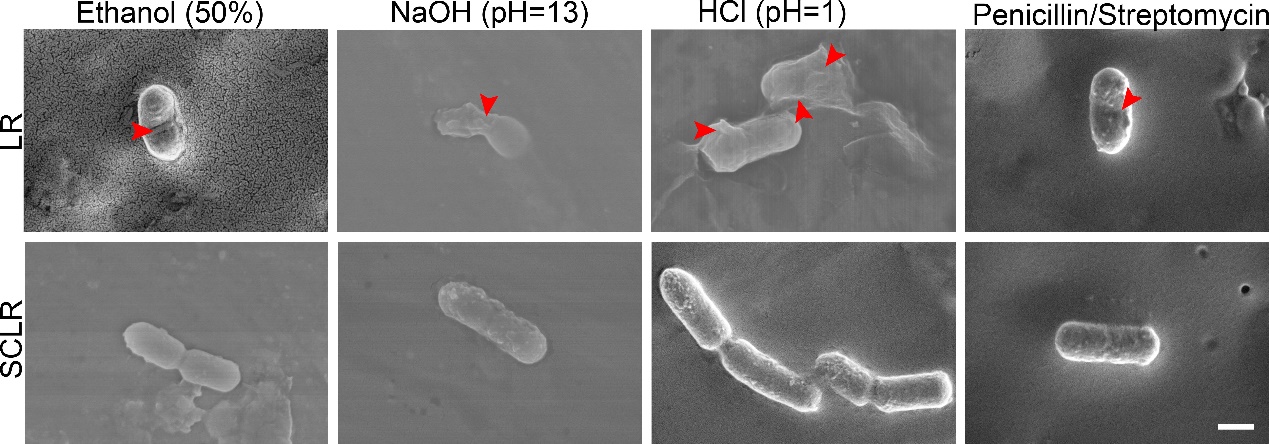


**Supplementary Fig. 5.** The SEM images of LR, SCLR, and OASCLR cultured in different environment (ethanol (50%, v/v), NaOH (pH=13), HCl (pH=1), and penicillin/streptomycin (10000 U/mL)) at 37 °C for 2 h. Scale bar, 0.5 μm.


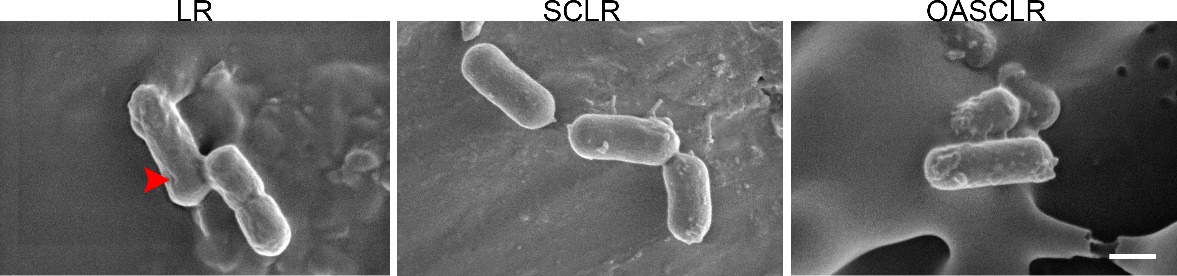


**Supplementary Fig. 6.** The SEM images of LR, SCLR, and OASCLR cultured in simulated pulmonary environment pH (pH=6) at 37 °C for 2 h. Scale bar, 0.5 μm.


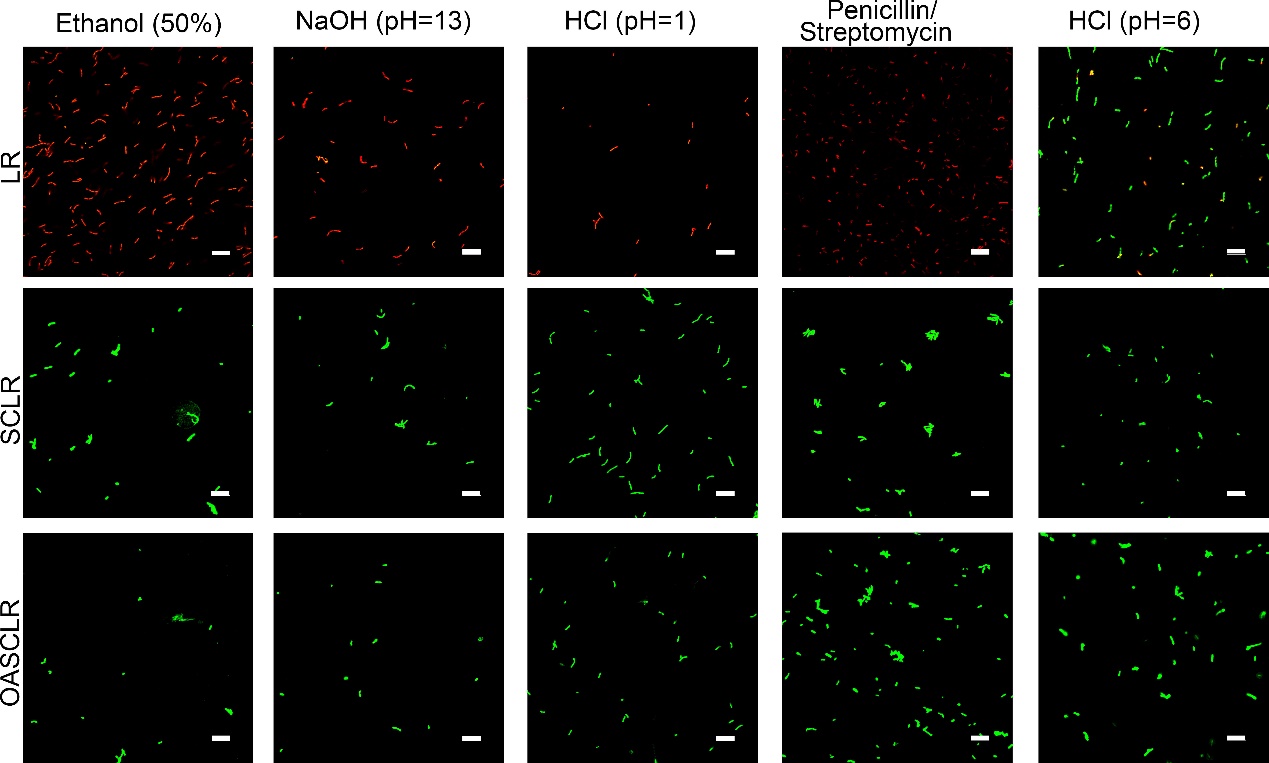


**Supplementary Fig. 7.** The images of bacterial live/dead staining of LR, SCLR, and OASCLR after culturing with different environment (ethanol (50%, v/v), NaOH (pH=13), HCl (pH=1), penicillin/streptomycin (10000 U/mL), and simulated pulmonary environment pH (pH=6)) at 37 °C for 2 h. SYTO 9 and propidium iodide (PI) were used to stain live and dead bacteria into green and red color, respectively. Scale bar, 10 μm.

The influence of different environments on the bacterial viability of LR, SCLR, and OASCLR was characterized by bacterial live/dead fluorescence staining in **Supplementary Fig. 7**. Many red fluorescence (dead bacteria) was presented in LR group after incubating with ethanol (50%, v/v), NaOH (pH=13), HCl (pH=1), and penicillin/streptomycin at 37 °C for 2 h. In contrast, most of green fluorescence (live bacteria) could be detected in SCLR and OASCLR groups. As for the bacterial viability of LR, SCLR, and OASCLR, only a few dead bacteria could be observed in LR group after incubating with simulated pulmonary environment pH (pH=6) at 37 °C for 2 h. The SCLR and OASCLR groups showed a great number of green fluorescence (live dead). These results suggested that the LR stability was enhanced after coating.


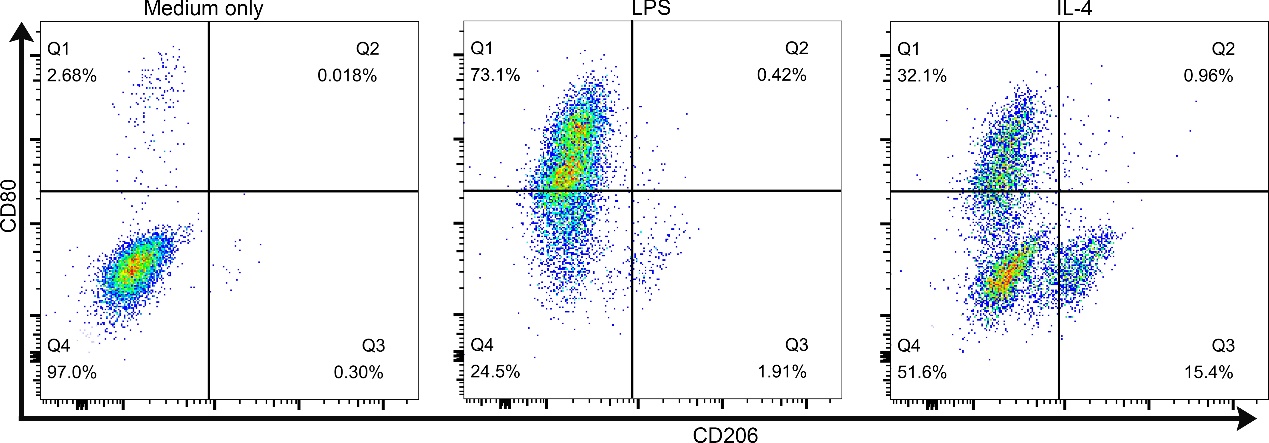


**Supplementary Fig. 8.** Flow cytometry results of CD206 (M2 marker) and CD80 (M1 marker) expression after 24 h treatment with medium only, LPS (100 ng/mL), and IL-4 (20 ng/mL).

CD206 and CD80 was chosen as M2 marker and M1 marker of macrophage, respectively. As shown in **Supplementary Fig. 8**, the expression of CD80 (M1 marker) and CD206 (M2 marker) was low in medium only group, suggesting that the original status of macrophages was M0-type phenotype. The expression of CD80^high^CD206^neg^ in medium only, LPS, and IL-4 groups was 2.68%, 73.1%, and 32.1%, respectively. The expression of CD206^high^CD80^neg^ in medium only, LPS, and IL-4 groups was 0.3%, 1.91%, and 15.4%, respectively. The ratio between CD80^high^CD206^neg^ and CD206^high^CD80^neg^ in medium only and LPS groups was 8.93 and 38.27, respectively. The result indicated that macrophages tended to polarize into M1-type macrophages after treating with LPS. On the other hand, the ratio between CD206^high^CD80^neg^ and CD80^high^CD206^neg^ in medium only and IL-4 groups was 0.11 and 0.47, respectively. The result suggested that macrophages tended to polarize into M2-type macrophages after treating with IL-4.


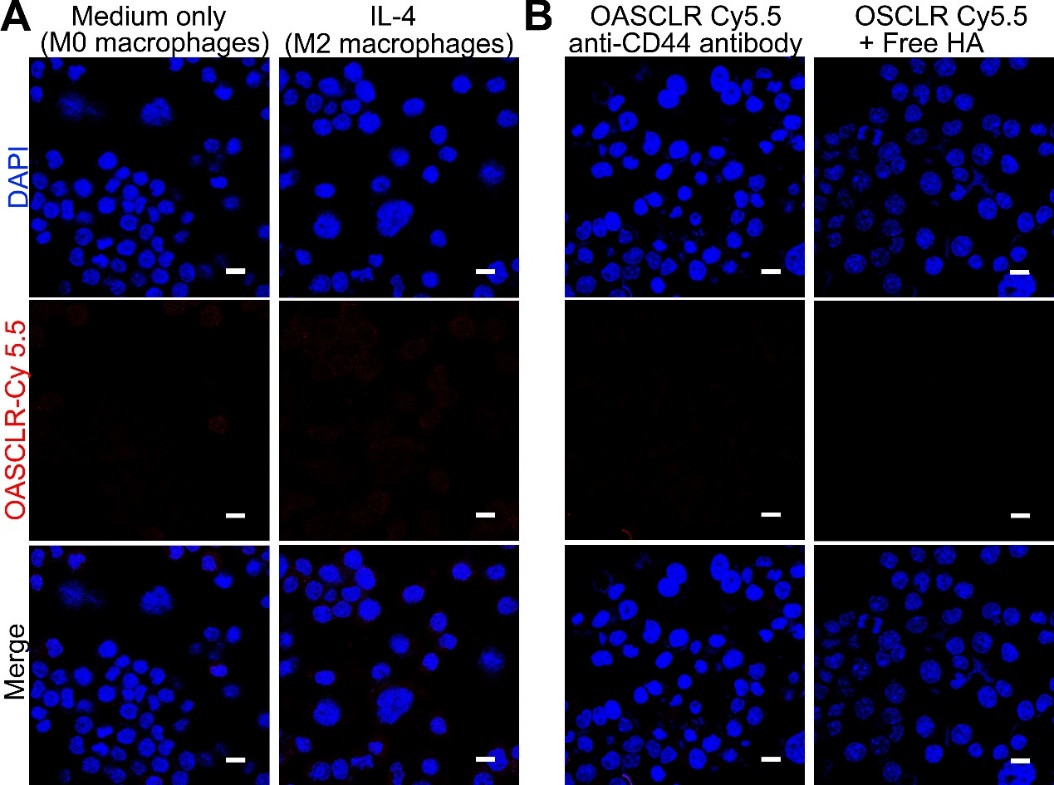


**Supplementary Fig. 9.** (**A**) Left: Confocal microscopy image of macrophages pre-treated for 24 h with culture medium, followed by 1 h incubation with OASCLR-Cy 5.5 (10^7^ CFU/mL). Right: Confocal microscopy image of macrophages pre-treated for 24 h with IL-4 (20 ng/mL), followed by 1 h incubation with OASCLR-Cy 5.5 (10^7^ CFU/mL). Hoechst 33342 was used for nuclei staining. Scale bar, 10 μm. (**B**) Left: The confocal microscopy image of macrophages pre-treated for 24 h with LPS (100 ng/mL), incubated with anti-CD44 antibody (500 μg/mL), followed by treatment with OASCLR-Cy 5.5 (10^7^ CFU/mL) for 1 h. Right: The confocal microscopy image of macrophages pre-treated for 24 h with LPS (100 ng/mL), followed by treatment with OSCLR-Cy 5.5 (10^7^ CFU/mL) for 1 h. OSCLR was OASCLR without HA. Hoechst 33342 was used for nuclei staining. Scale bar, 10 μm.


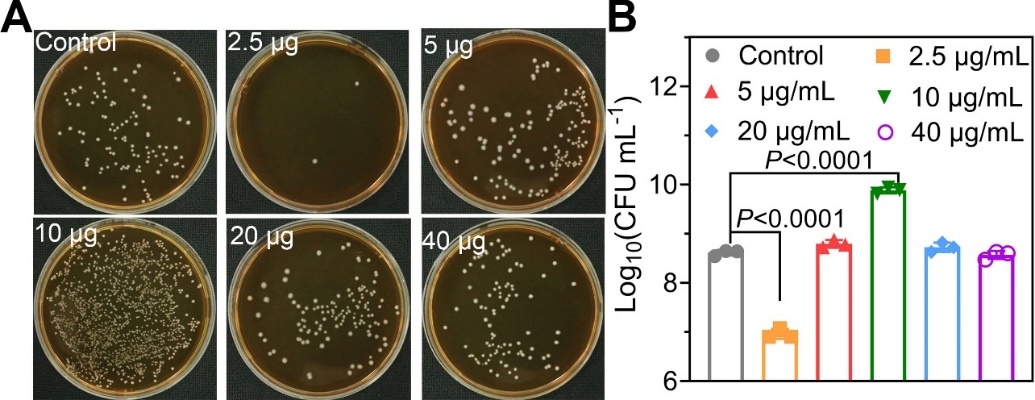


**Supplementary Fig. 10.** (**A**) The spread plate pictures of LR after incubating with different concentrations of ononin (0 μg/mL, 2.5 μg/mL, 5 μg/mL, 10 μg/mL, 20 μg/mL, and 40 μg/mL) for 24 h. (**B**) The CFU value of LR after incubating with different concentrations of ononin (0 μg/mL, 2.5 μg/mL, 5 μg/mL, 10 μg/mL, 20 μg/mL, and 40 μg/mL). Data are presented as mean ± standard deviation (SD). Data was analyzed by one-way ANOVA with multiple comparison test. (n = 3 biologically independent samples).

**Supplementary Fig. 10** presented the influence of different concentrations of ononin on the growth of LR. The ononin (10 μg/mL) could enhance the growth of LR.


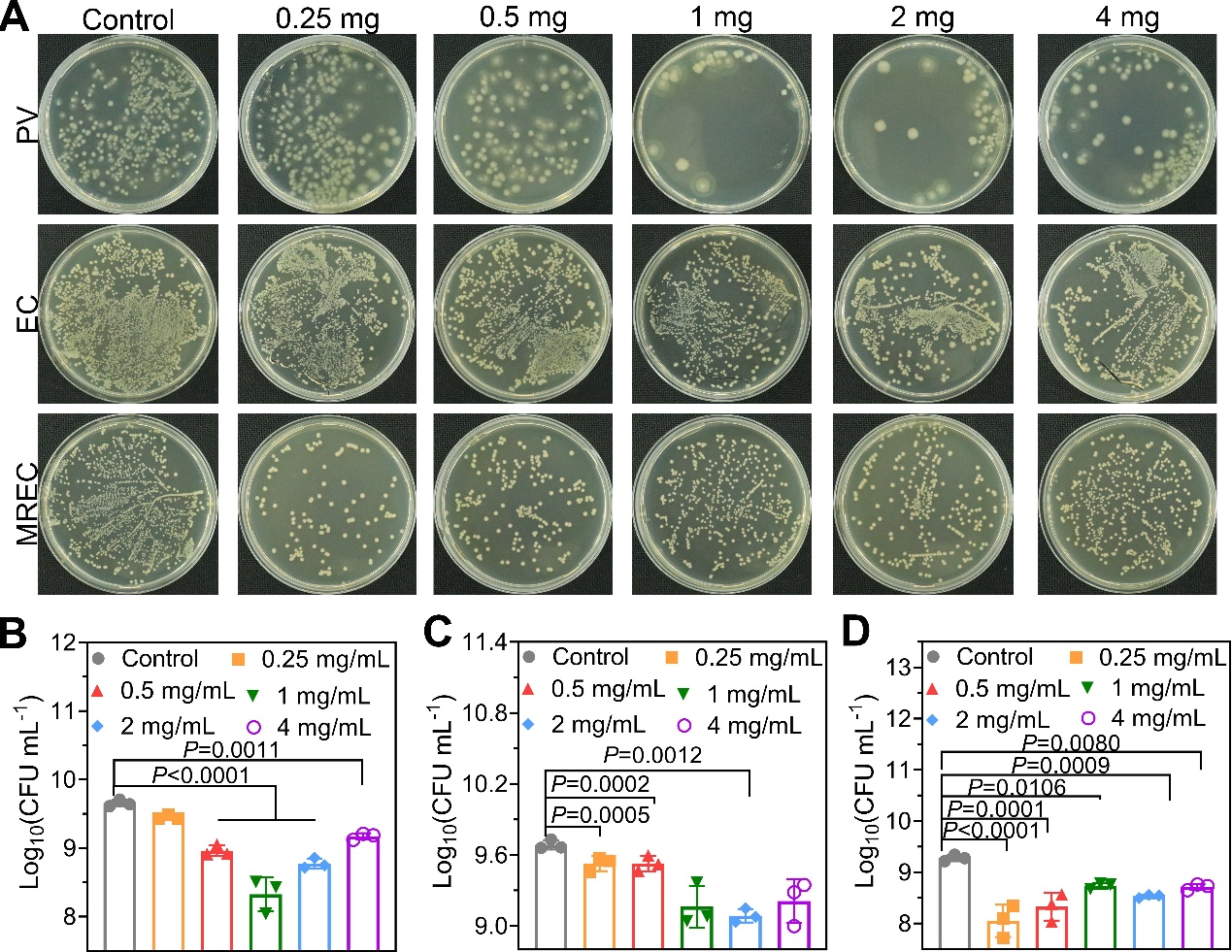


**Supplementary Fig. 11.** (**A**) The spread plate pictures of PV, EC, and MREC after treating for 24 h with different concentrations of ononin (0 mg/mL, 0.25 mg/mL, 0.5 mg/mL, 1 mg/mL, 2 mg/mL, and 4 mg/mL). The CFU value of (**B**) PV, (**C**) EC, and (**D**) MREC after treating for 24 h with different concentrations of ononin (0 mg/mL, 0.25 mg/mL, 0.5 mg/mL, 1 mg/mL, 2 mg/mL, and 4 mg/mL). **B-D.** Data are presented as mean ± standard deviation (SD). Data was analyzed by one-way ANOVA with multiple comparison test. (n = 3 biologically independent samples).

As shown in **Supplementary Fig. 11**, the different concentration of ononin (0.25 mg/mL, 0.5 mg/mL, 1 mg/mL, 2 mg/mL, and 4 mg/mL) showed some antibacterial performance against *Proteusbacillus vulgaris* (PV), multi-resistant *Escherichia coli* (MREC), and *E. coli* (EC).


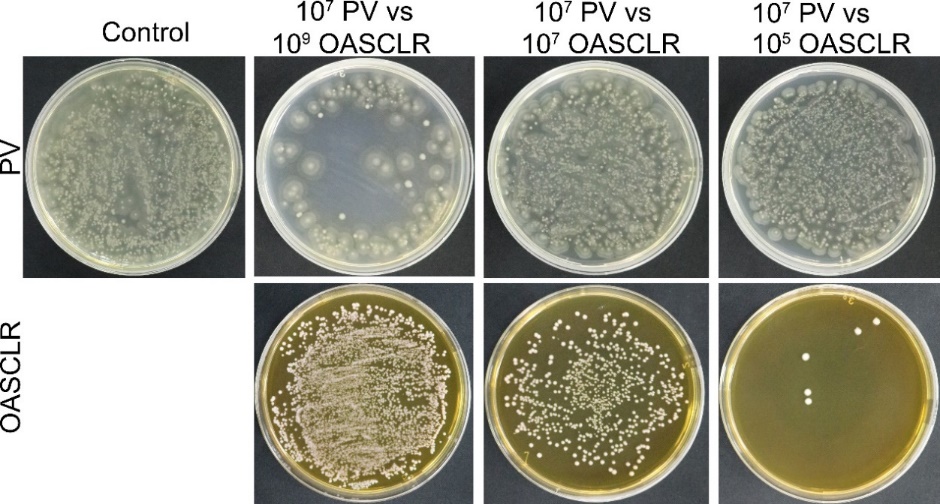


**Supplementary Fig. 12.** The spread plate pictures of PV and OASCLR after incubating PV with different concentration of OASCLR for 24 h.


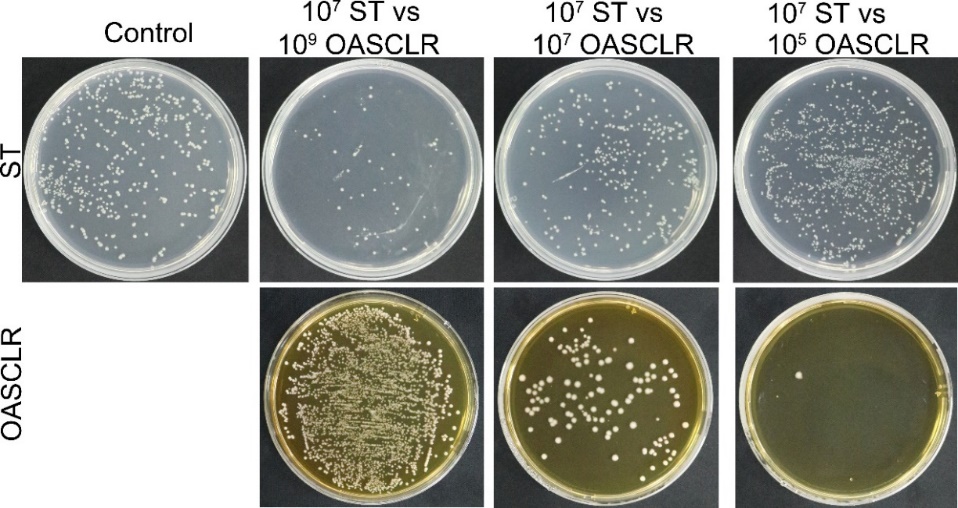


**Supplementary Fig. 13.** The spread plate pictures of *Salmonella typhimurium* (ST) and OASCLR after incubating ST with different concentration of OASCLR for 24 h.


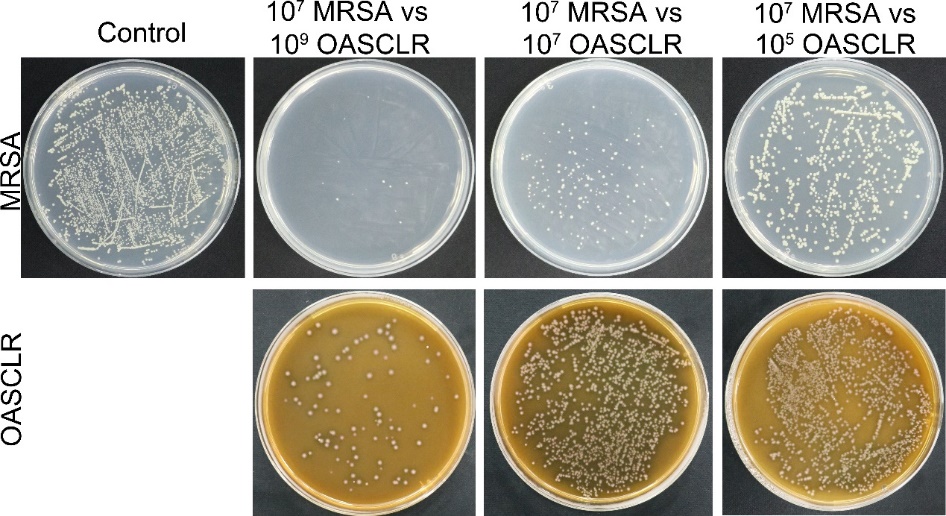


**Supplementary Fig. 14.** The spread plate pictures of MRSA and OASCLR after incubating MRSA with different concentration of OASCLR for 24 h.


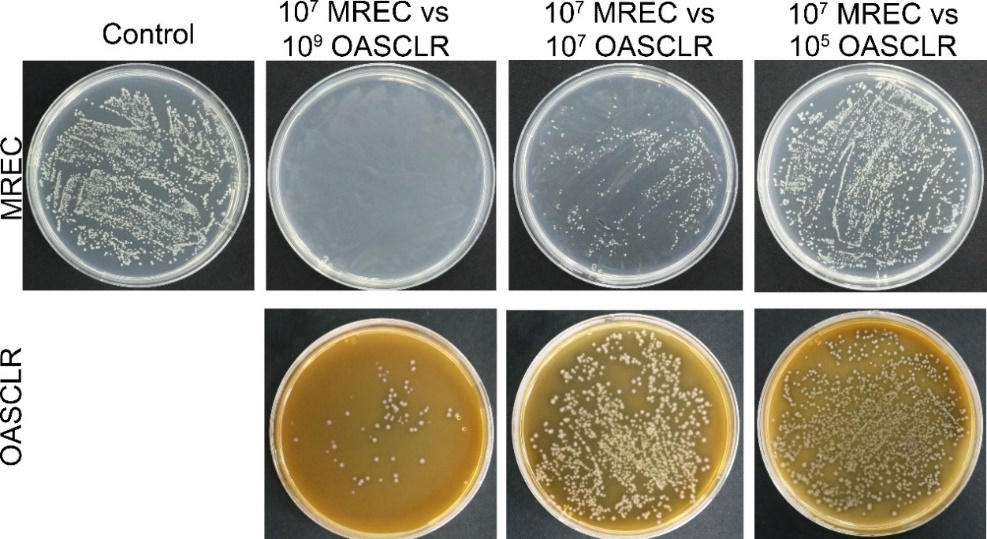


**Supplementary Fig. 15.** The spread plate pictures of MREC and OASCLR after incubating MREC with different concentration of OASCLR for 24 h.


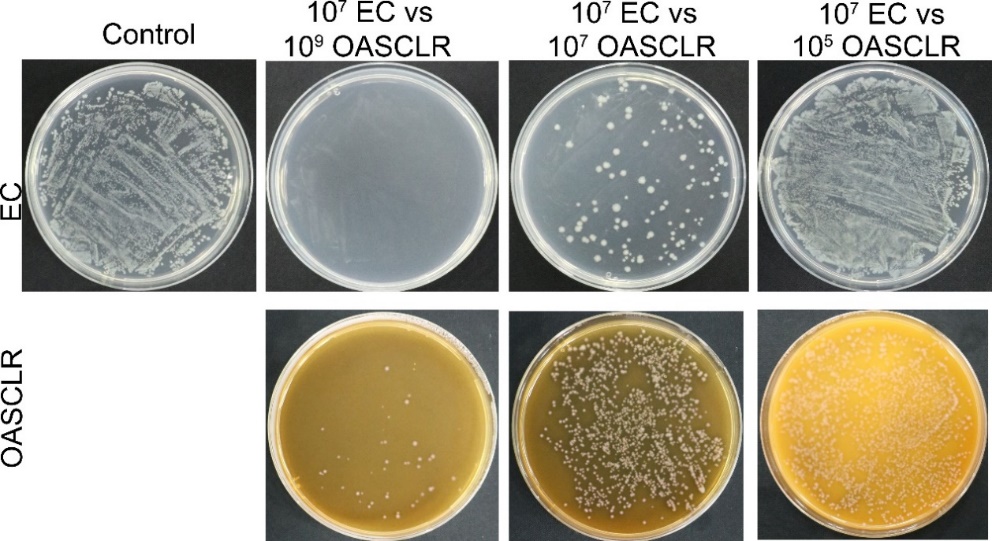


**Supplementary Fig. 16.** The spread plate pictures of EC and OASCLR after incubating EC with different concentration of OASCLR for 24 h.


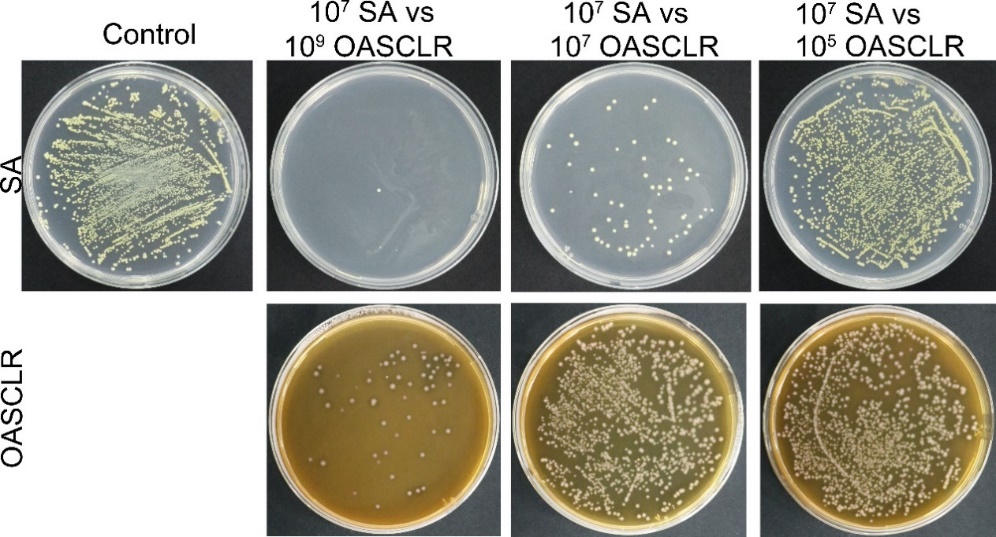


**Supplementary Fig. 17** The spread plate pictures of SA and OASCLR after incubating SA with different concentration of OASCLR for 24 h.


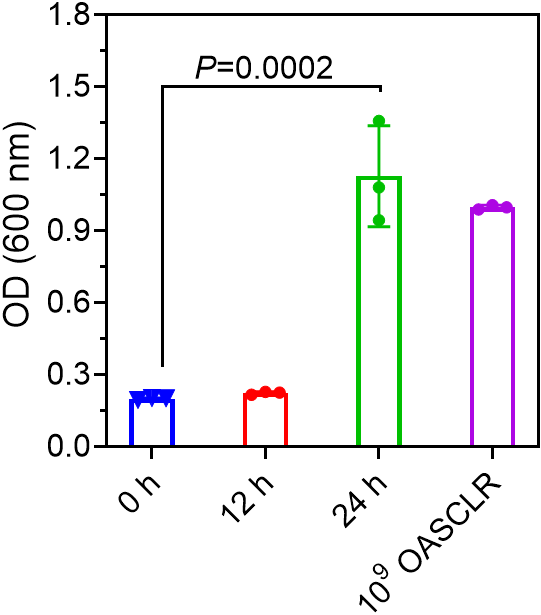


**Supplementary Fig. 18.** The growth condition of bacteria, and the initial bacterial number of OASCLR was 10^7^ CFU/mL. Data are presented as mean ± standard deviation (SD). Data was analyzed by one-way ANOVA with multiple comparison test. (n = 3 biologically independent samples).


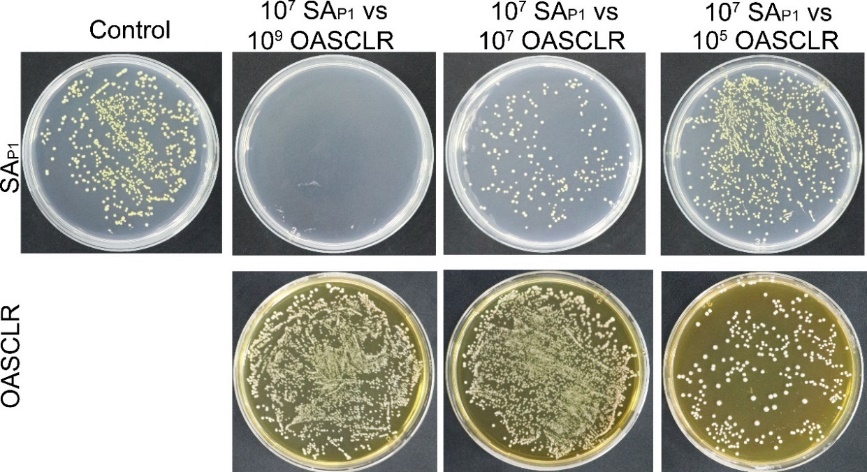


**Supplementary Fig. 19.** The spread plat pictures of SA_P1_ and OASCLR after incubating SA_P1_ with different concentration of OASCLR for 24 h.


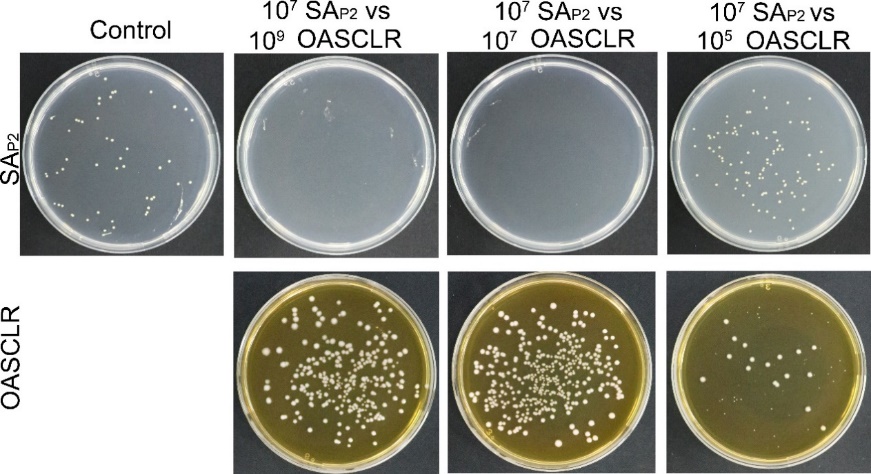


**Supplementary Fig. 20.** The spread plat pictures of SA_P2_ and OASCLR after incubating SA_P2_ with different concentration of OASCLR for 24 h.


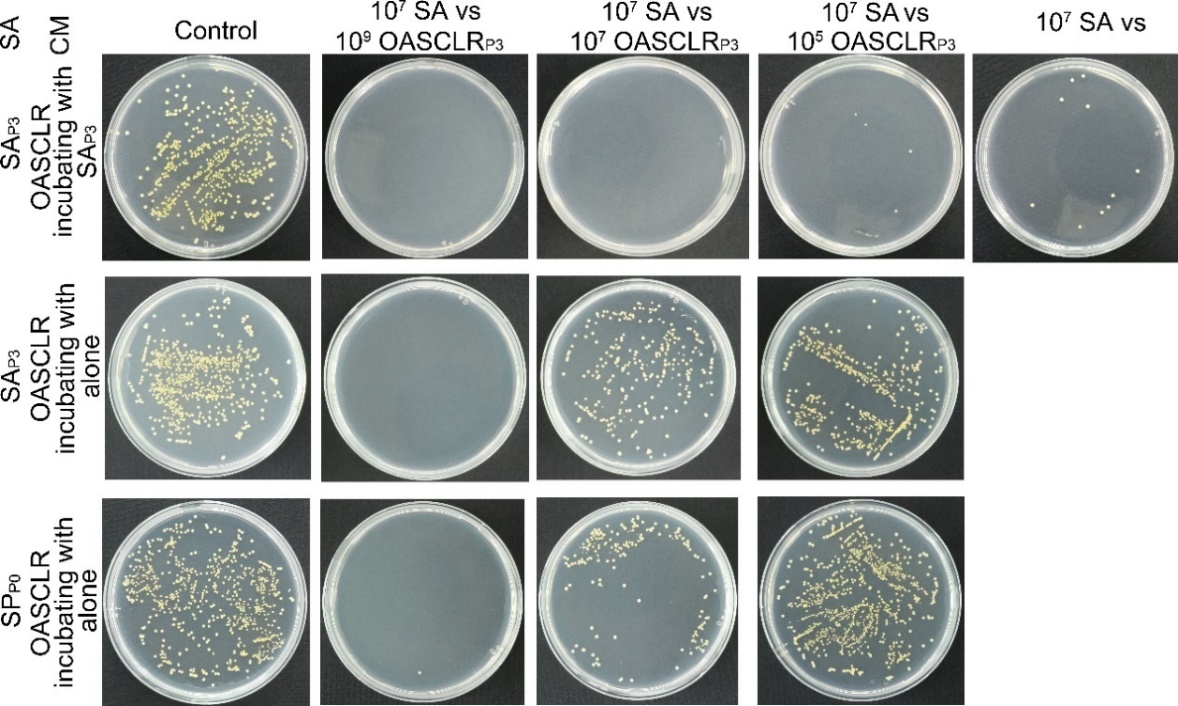


**Supplementary Fig. 21.** The spread plat pictures of SA_P3_ and SA_P0_ after treating with different CM.


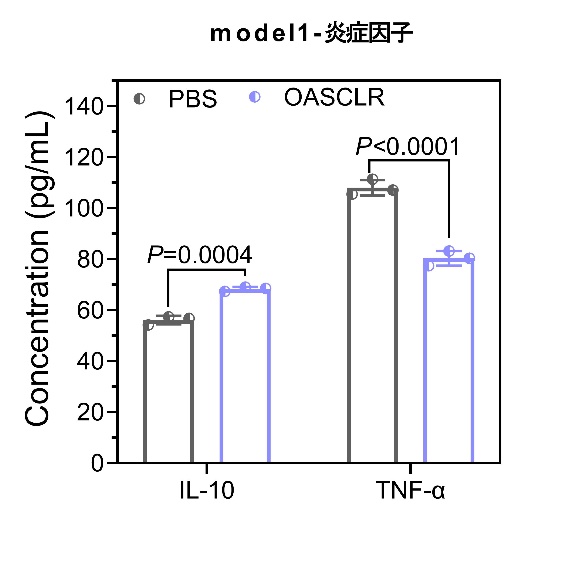


**Supplementary Fig. 22.** The concentration of protein IL-10 and protein TNF-α of two groups (PBS and OASCLR) analyzed by ELISA assay. Data are presented as mean ± standard deviation (SD). Data was analysed by two-way ANOVA with multiple comparison test. (n = 3 biologically independent samples).


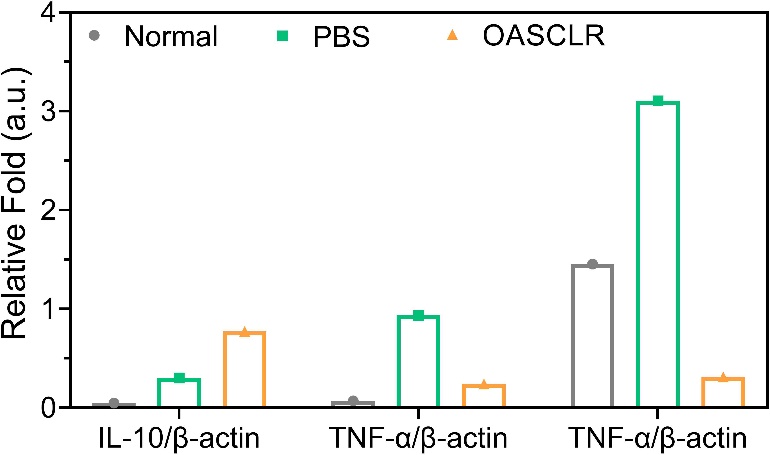


**Supplementary Fig. 23.** Quantitative analysis of the expression content of TNF-α and IL-10 proteins by WB. (n = 1 biologically independent samples).


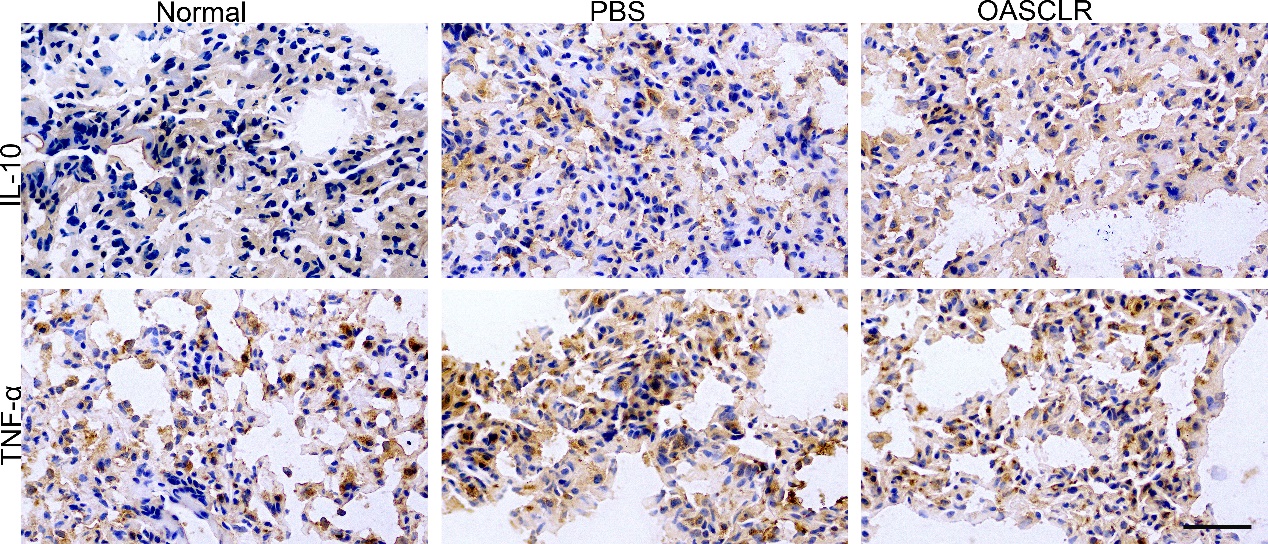


**Supplementary Fig. 24.** Immunohistochemical staining of IL-10 and TNF-α in the mice lung tissue. Scale bar, 50 μm.


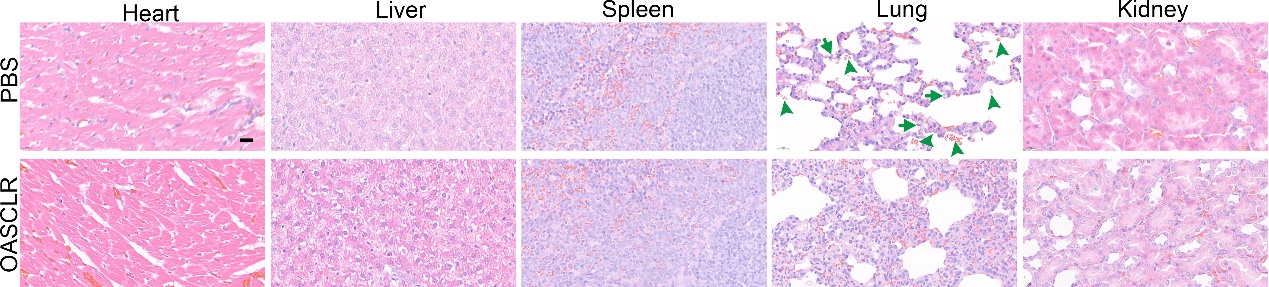


**Supplementary Fig. 25.** Histological examinations of H&E-stained major organ sections, including heart, liver, spleen, lung, and kidney. The inflammatory cells and red blood cells were marked by green arrow and arrowhead, respectively. Scale bar, 20 μm.


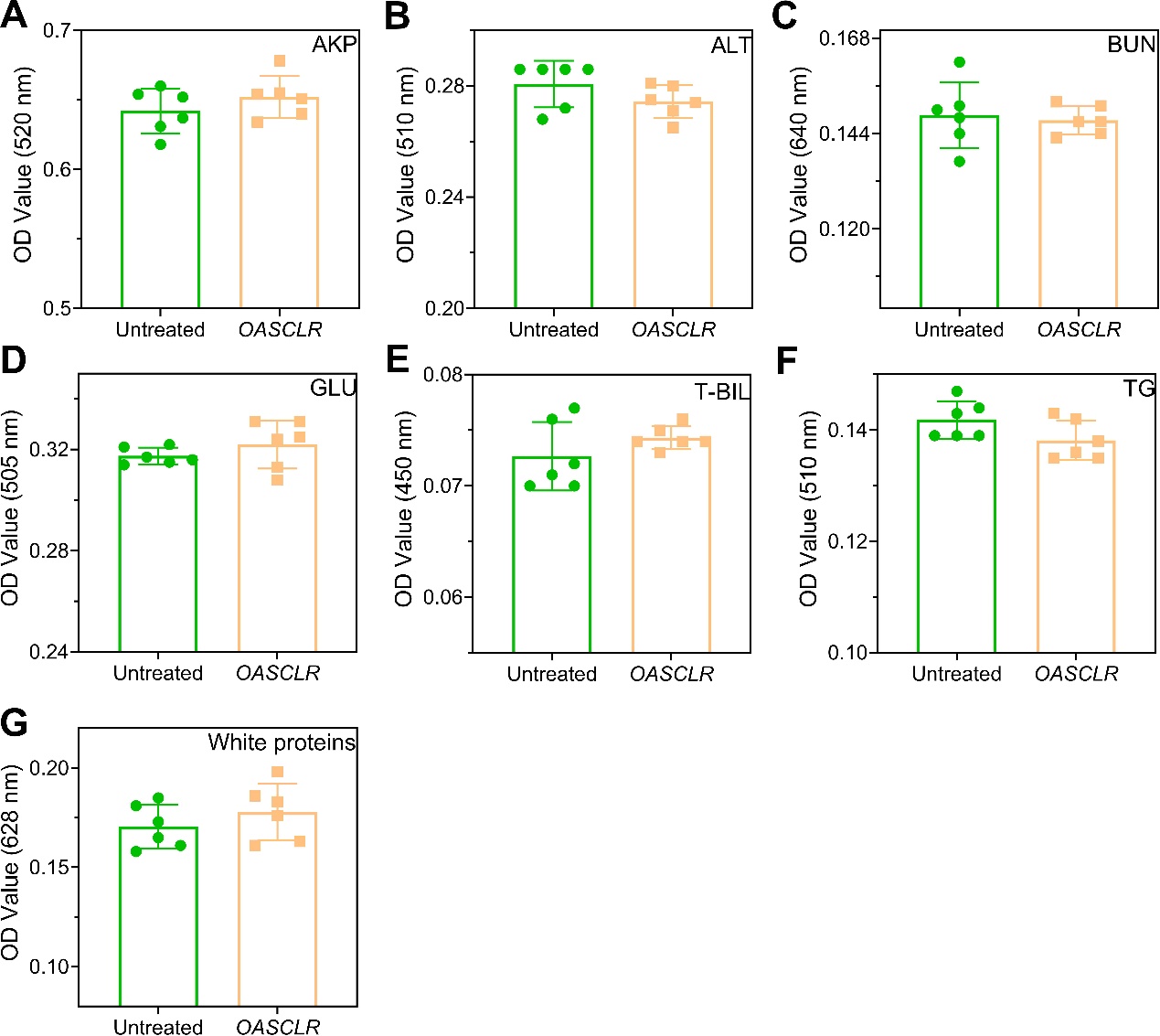


**Supplementary Fig. 26.** Evaluation of the hepatotoxicity and nephrotoxicity of untreated and OASCLR groups (according to the serum levels of AKP, ALT, BUN, GLU, T-BIL, TG, and white proteins) after different treatments. (n = 6 biologically independent samples).


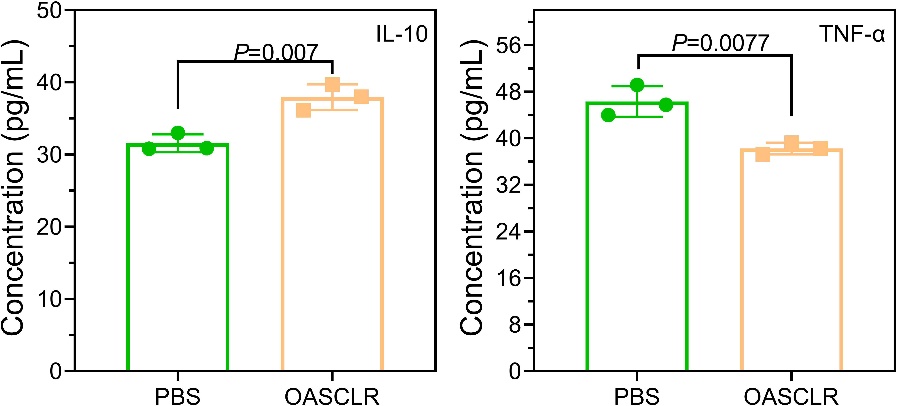


**Supplementary Fig. 27.** The concentration of IL-10, and TNF-α of two groups (PBS and OASCLR) analyzed by ELISA assay. Data are presented as mean ± standard deviation (SD). Data was analyzed by t test. (n = 3 biologically independent samples).


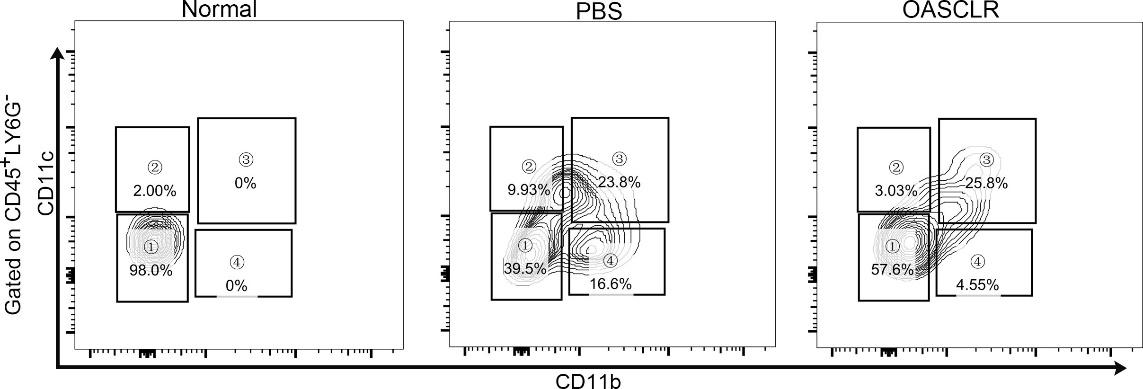


**Supplementary Fig. 28.** Characterization of pro-inflammatory monocytes/alveolar macrophages in lung tissue of Normal, PBS, and OASCLR groups. Subsets (gated on CD45^+^LY6G^-^) were characterized based on CD11b and CD11c expression as pro-inflammatory monocytes (CD11b^high^, CD11c^neg^) and alveolar macrophages (CD11c^high^, CD11b^neg^).


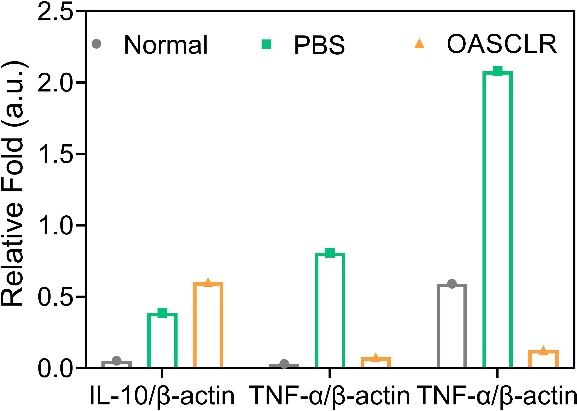


**Supplementary Fig. 29.** Quantitative analysis of the expressive content of TNF-α and IL-10 proteins by WB. (n = 1 biologically independent samples).


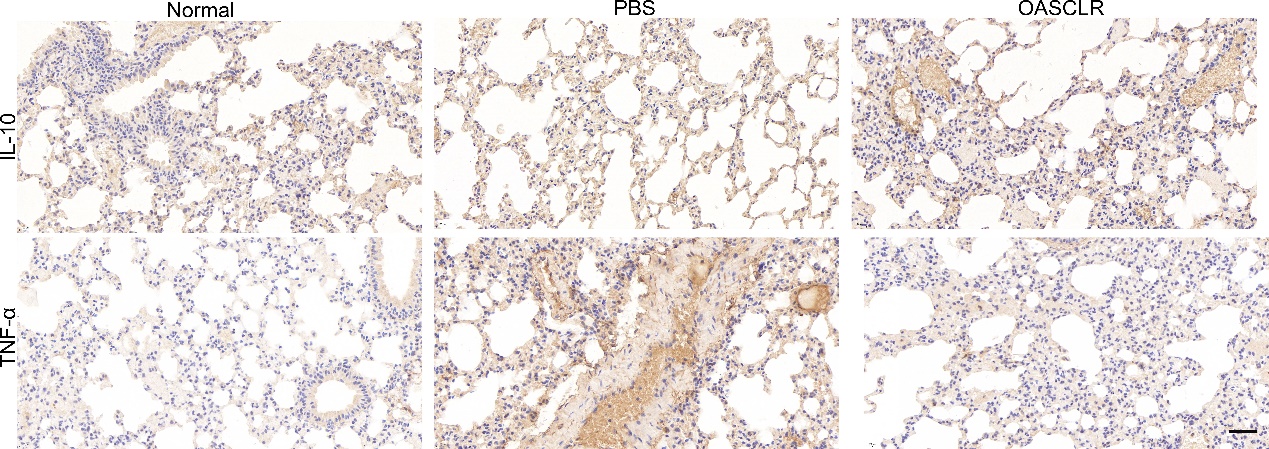


**Supplementary Fig. 30.** Immunohistochemical staining of IL-10 and TNF-α in the mice lung tissue. Scale bar, 50 μm.


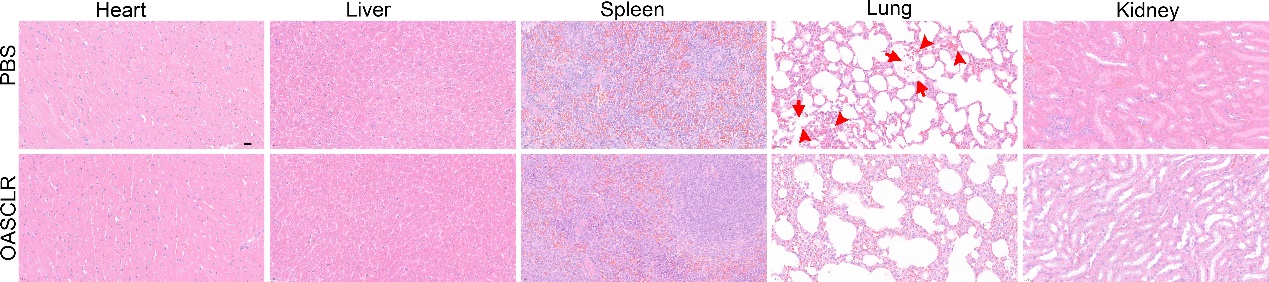


**Supplementary Fig. 31.** Images of H&E staining for major organs, including heart, liver, spleen, lung, and kidney. (The inflammatory cells and fibrin strains were marked by red arrowhead and arrow, respectively.) Scale bar, 20 μm.


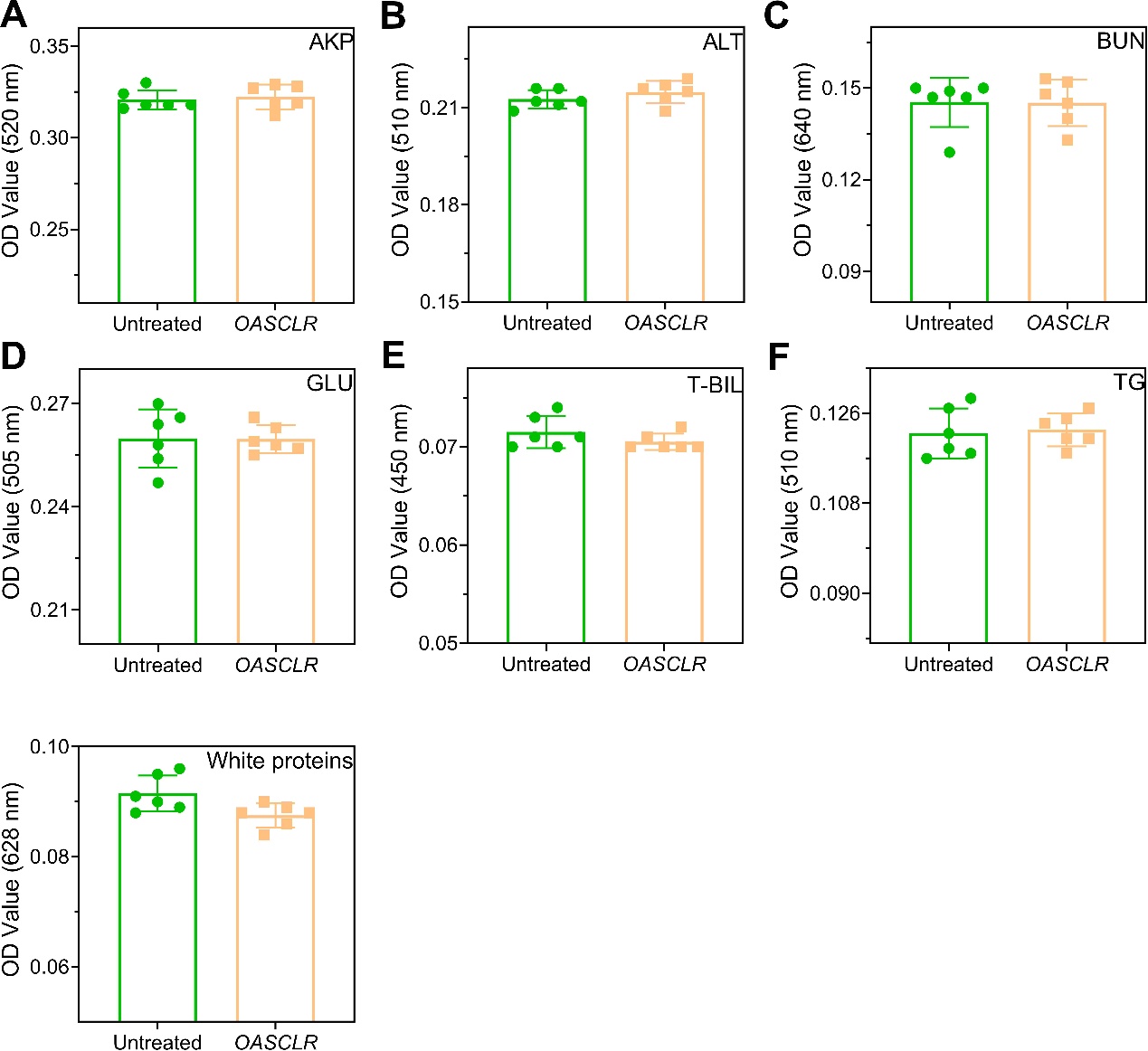


**Supplementary Fig. 32.** Evaluation of the hepatotoxicity and nephrotoxicity of untreated and OASCLR groups (according to the serum levels of AKP, ALT, BUN, GLU, T-BIL, TG, and white proteins) after different treatments. (n = 6 biologically independent samples).

| **Supplementary Table 1.** Antimicrobial activity of LR and OASCLR against PV, ST, MRSA, MREC, EC, and SA. | | | | | | |
| --- | --- | --- | --- | --- | --- | --- |
| Name | MBC*(CFU/mL) | | | | | |
|  | PV | ST | MRSA | MREC | EC | SA |
| LR | 1.25×10^8^ | 1×10^9^ | 1×10^9^ | 1.95×10^6^ | 1.95×10^6^ | 2.5×10^8^ |
| OASCLR | 2×10^9^ | 4×10^9^ | 1×10^9^ | 1.25×10^8^ | 1.25×10^8^ | 5×10^8^ |

**Supplementary Table 1.** Antimicrobial activity of LR and OASCLR against PV, ST, MRSA, MREC, EC, and SA. MBC* is defined as the concentration of sample, which could lead the value of CFU to becoming zero. The concentration of LR and OASCLR was chosen as MBC when the spread plate appeared several bacteria.

**REFERENCES**

1. Lee Y, Sugihara K and Gillilland MG *et al.* Hyaluronic acid-bilirubin nanomedicine for targeted modulation of dysregulated intestinal barrier, microbiome and immune responses in colitis. *Nat Mater* 2020; **19**: 118-26.

2. Fu J, Liu X and Tan L *et al.* Modulation of the mechanosensing of mesenchymal stem cells by laser-induced patterning for the acceleration of tissue reconstruction through the Wnt/beta-catenin signaling pathway activation. *Acta Biomater* 2020; **101**: 152-67.
